# A Pumpless, High-Throughput Microphysiological System to Mimic Enteric Innervation of Duodenal Epithelium and the Impact on Barrier Function

**DOI:** 10.1002/adfm.202409718

**Published:** 2024-09-03

**Authors:** Kyla N. Kaiser, Jessica R. Snyder, Ryan A. Koppes, Abigail N. Koppes

**Affiliations:** Department of Chemical Engineering, Northeastern University, Boston, MA 02155, USA; Department of Bioengineering, Northeastern University, Boston, MA 02155, USA; Department of Chemical Engineering, Northeastern University, Boston, MA 02155, USA; Department of Bioengineering, Northeastern University, Boston, MA 02155, USA; Department of Chemical Engineering, Northeastern University, Boston, MA 02155, USA; Department of Bioengineering, Northeastern University, Boston, MA 02155, USA; Department of Biology, Northeastern University, Boston, MA 02155, USA

**Keywords:** co-culture, enteric nervous system, high throughput, intestine, microphysiological system, organ-chip

## Abstract

Enteric neurons are critical in maintaining organ homeostasis within the small intestine, and their dysregulation are implicated in gastrointestinal disorders and neurodegenerative diseases. Most in vitro models lack enteric innervation, limiting basic discovery and disease modeling research. Here, a high-throughput 3D microphysiological system (MPS), or organ chip is presented that supports a primary epithelial monolayer interfacing directly with encapsulated primary enteric neurons. The device features twelve 3D MPSs per device and gravity-driven flow via a laboratory rocker to induce biomimetic shear stress on the epithelium culture and provide continuous nutrient presentation. Intestinal and neural tissue exhibited expected morphologies. Neural gene upregulation in the epithelium suggests RNA contamination from proximal enteric neurons extending neurites toward the epithelial monolayer. With the enteric nervous system (ENS), barrier integrity significantly increased for both TEER and permeability assays, a 1.25-fold greater resistance and 10% lower permeability as compared to epithelium cultured alone. The presence of the ENS resulted in a significant (1.4-fold) reduction in epidermal growth factor (EGF). Additionally, several key epithelial genes are compared between duodenal tissue and epithelial monolayers with and without neurons present. Results demonstrated changes in cytokine gene expression and WNT pathways, highlighting innervation is essential to create more biomimetic and physiologically relevant in vitro models.

## 1. Introduction

The gastrointestinal (GI) tract forms a selectively permeable barrier, allowing nutrient transport into the host while keeping pathogens out. The gut is host to millions of harmful and symbiotic microorganisms that the intestinal epithelium must keep separate from the inner circulatory system. The integrity of this epithelial barrier is essential for systemic health. A compromised intestinal barrier is implicated in several GI disorders due to bacteria and toxins infiltrating the tissue and causing inflammation. These disorders include the discernible irritable bowel syndrome (IBS) and Inflammatory Bowel Disease (IBD), and are also comorbid with several diseases including depression and hypertension, suggesting gut health impacts the entire body.^[1–4]^ The underlying mechanisms of GI disorders remain elusive due to the complexity of entire organisms, especially considering the interplay between cognitive and gut health during in vivo investigations. Therefore, a reductionist approach utilizing in vitro models is necessary to understand the regulation of barrier function and the impact the gut can have on human systemic health.

Gut microphysiological systems (MPSs), or organ chips, have been developed with various materials and architectures to add physiological relevance, such as human tissue, inflammatory compounds, shear stress to model peristalsis, compositions of the microbiome, and villi-like structures.^[5–10]^ Due to their advantages over in vivo models, these MPSs are used to study broad areas, from drug delivery to disease progression.^[11,12]^ The expanded complexity of gut-on-a-chip systems, including physiological components such as shear stresses via medium perfusion, have shown faster differentiation and increased mucus production compared to traditional 2D culture methods.^[8,13]^ These MPSs also support analysis in real-time, including standard light microscopy and the ability to assess barrier function and permeability, which are especially relevant in modeling epithelial dysfunction such as in IBD. Transepithelial electrical resistance (TEER) and apparent permeability measurements of fluorescent molecule diffusion are non-destructive methods often used to assess barrier integrity of live epithelial monolayers on chip.^[8,14,15]^ The health of these epithelial barrier properties are pivotal for studying microbiome interactions and drug absorption.^[16,17]^ These techniques are easily implemented in Transwell cultures, however the difficulty and cost of integrating sensing modalities of barrier function, especially TEER, limits broader adoption of MPSs.

Current gut epithelium MPSs require cell lines, isolated primary cells, or induced stem cells. The human colorectal adenocarcinoma cancer cell line, Caco-2, is frequently used as an epithelium model with maturation leading to tight junction formation. However, Caco-2 cells have limitations as they only comprise the absorptive enterocyte phenotype, lacking the heterogeneous population found in vivo.^[18]^ The field is moving to primary epithelium cell models, often seeding monolayers of epithelial cells isolated from expandable intestinal epithelial organoids which contain intestinal stem cells and epithelial cell subtypes responsible for neural, immune, and microbial cell interactions. Specifically, these diverse populations include absorptive enterocytes, mucus-producing goblet cells, and secretory enteroendocrine and Paneth cells.^[19]^ Primary epithelium models are only obtainable through rodent tissue or human biopsy samples. However, in the dish, these populations proliferate easily, allowing sizable stocks of the cells to be cultured or cryopreserved for up to several years.^[20]^ These heterogeneous epithelium populations more closely resemble the human gut than traditional immortalized cancer cell lines and are needed to explore the role of the autonomic nervous system (ANS) of barrier function.

The degree and synaptic organization of the enteric nervous system (ENS) on the function of each of these epithelial populations is not fully understood. Sensations and coordination of gastrointestinal (GI) activities are facilitated by the underlying neurons of the gut, the ENS, as well as branches of the autonomic nervous system composed of plexi, ganglia, spinal cord, and cranial nerves. Greater in total neuron numbers than the spinal cord, enteric neurons inform the epithelium’s proliferation through epidermal growth factor (EGF) signaling in enterocytes, the most abundant epithelial subtype.^[21]^ The enteric nervous system further aids in preventing microbial infection by promoting goblet cell mucus production, resulting in a thicker, stronger barrier. Enteric neurons also play a role in immunity, producing cytokines (IL-6, IL-18)^[22,23]^ as well as sensing (TNF-*α*, TGF-*β*, IL-4) inflammatory cytokines.^[24]^ Epithelial-enteric neuron communication has been found to occur through soluble neuronal mediators in addition to direct synapsing onto enteroendocrine cells.^[25]^ One example of paracrine signaling in the gut occurs through Vasoactive Intestinal Peptide (VIP) released by VIPergic enteric neurons. VIP promotes proliferation of the epithelial cells, improves the barrier, and increases secretion.^[26,27]^ Conversely, acetylcholine (Ach), released by cholinergic enteric neurons, increases permeability and decreases proliferation of the epithelium, suggesting that an imbalance between these two neurotransmitters can lead to barrier dysfunction.^[28,29]^

In addition to incorporating enteric neurons within our model system, we engineered a new MPS that addressed many of the current limitations that plague gut-chip designs: low sample throughput, high media consumption, and reliance on syringe pumps along with their required fluidic connectors to induce shear. Our MPS design supports primary epithelial monolayers, 3D enteric neuron encapsulation, and up to twelve independent samples all within a standard tissue culture plate footprint (≈85 × 127 mm). Uniform pulsatile flow/shear across each sample was accomplished with media reservoirs that support pumpless, gravity-driven flow when placed on a laboratory rocker to induce physiologically relevant shear stresses. The device was assembled with a glass base allowing for high resolution microscopy through the entire height of the MPS so that both the epithelial and neuronal populations can be monitored during the experimental time course and analyzed for endpoint immunohistochemical characterization.

We hypothesized the presence of enteric neurons would alter the epithelial differentiation and maturation timeframe compared to epithelial-only cultures. Using our custom MPS system, we studied the impact of enteric neurons on the epithelial barrier stability with permeability assays, TEER and lucifer yellow diffusion. ELISA assays and epithelial RNA sequencing were carried out as steps toward understanding the underlying biological mechanisms of ENS regulated barrier function in vitro and comparing culture gene expression to duodenal tissue. Our results indicate that enteric neurons positively influence epithelial barrier strength, alter growth factor signaling, and change gene expression. These results highlight the importance of further investigating the role of innervation when developing biomimetic MPS devices.

## 2. Results and Discussion

### 2.1. Co-Cultures of Epithelial Cells and Enteric Neurons are Supported by a Cost Effective, Pumpless, High Throughput MPS

**Figure 1.**
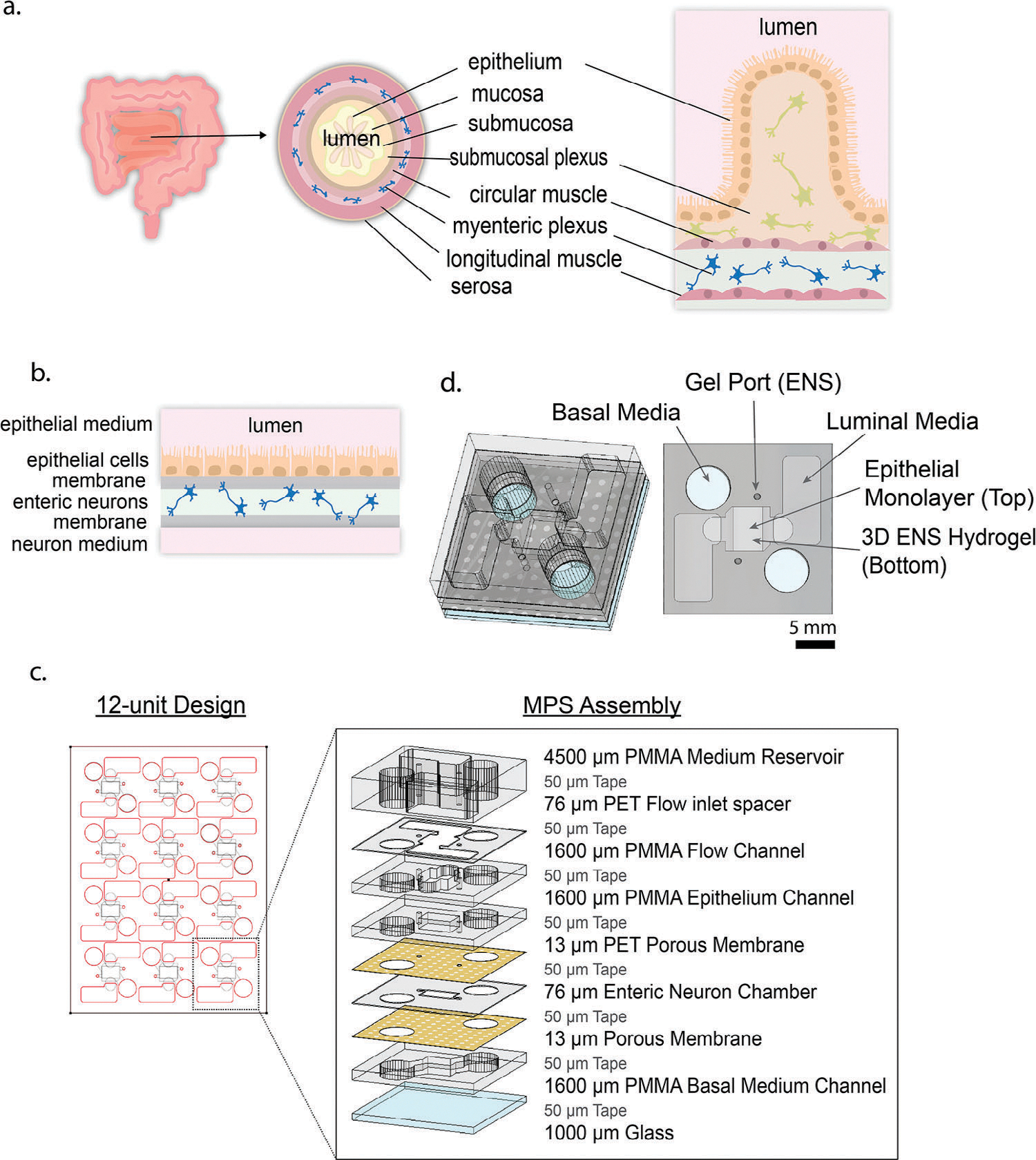
Chip designed to recapitulate the 3D small intestine into a simplified culture system. a) Cross sections of the bulk small intestine, simplified small intestine anatomy, and b) our reductionist model of the innervated small intestine. c) The 12-unit design fits within the dimensions of a standard 12 plate while keeping each 3D MPS independent for up to four experiments in parallel, in triplicate. The individual MPS comprises laser-cut thermoplastics (PET track etched membrane and PMMA spacers) bonded layer-by-layer with a 3M adhesive tape onto a glass slide. An exploded view of the chip layers and thicknesses. d) An orthogonal and straight on assembled version is displayed to highlight cell seeding ports as well as media wells for both the apical and basal chambers.

We developed a new innervated microphysiological system (MPS) of primary duodenal epithelium to investigate the role of enteric neurons on primary epithelial cell permeability (Figure 1a,b). This platform represents a departure from traditional MPSs fabricated via PDMS which are limited in the design/redesign^[30]^ and often cost prohibitive at $150–500 per design.^[8]^ The addition of neurons is critical for developing more complex and biomimetic organ chip devices, especially as they become the standard for studying developmental biology, drug delivery, and disease progression.^[31]^ The 12-unit MPSs are fabricated using laser-cut thermoplastic layers, a method previously described^[8,32]^ (Figure 1c). Polymethyl methacrylate (PMMA) and polyethylene terephthalate (PET) layers facilitate an oxygen impermeable environment,^[33,34]^ and the 12-sample chip can be produced in under a couple of hours for $21 dollars, or less than $2 per unit/sample (Figure 1b,c). The scaled-up device supports 12 co-culture chambers on a single 76 mm by 101 mm chip which fits well within a standard well plate footprint with dimensions around 127 mm by 85 mm. This platform addresses several of the current challenges in the robustness, reproducibility, and reliability of organ-chip platforms. The MPS features a top chamber on a permeable membrane allowing epithelial monolayer adhesion and polarization shown as a cross section (Figure 1b). There is a chamber supporting 3D culture below the membrane where enteric neurons were seeded within a Matrigel and collagen solution (Figure 1c). Figure 1c shows an exploded view of a single MPS and its culture setup.

**Figure 2.**
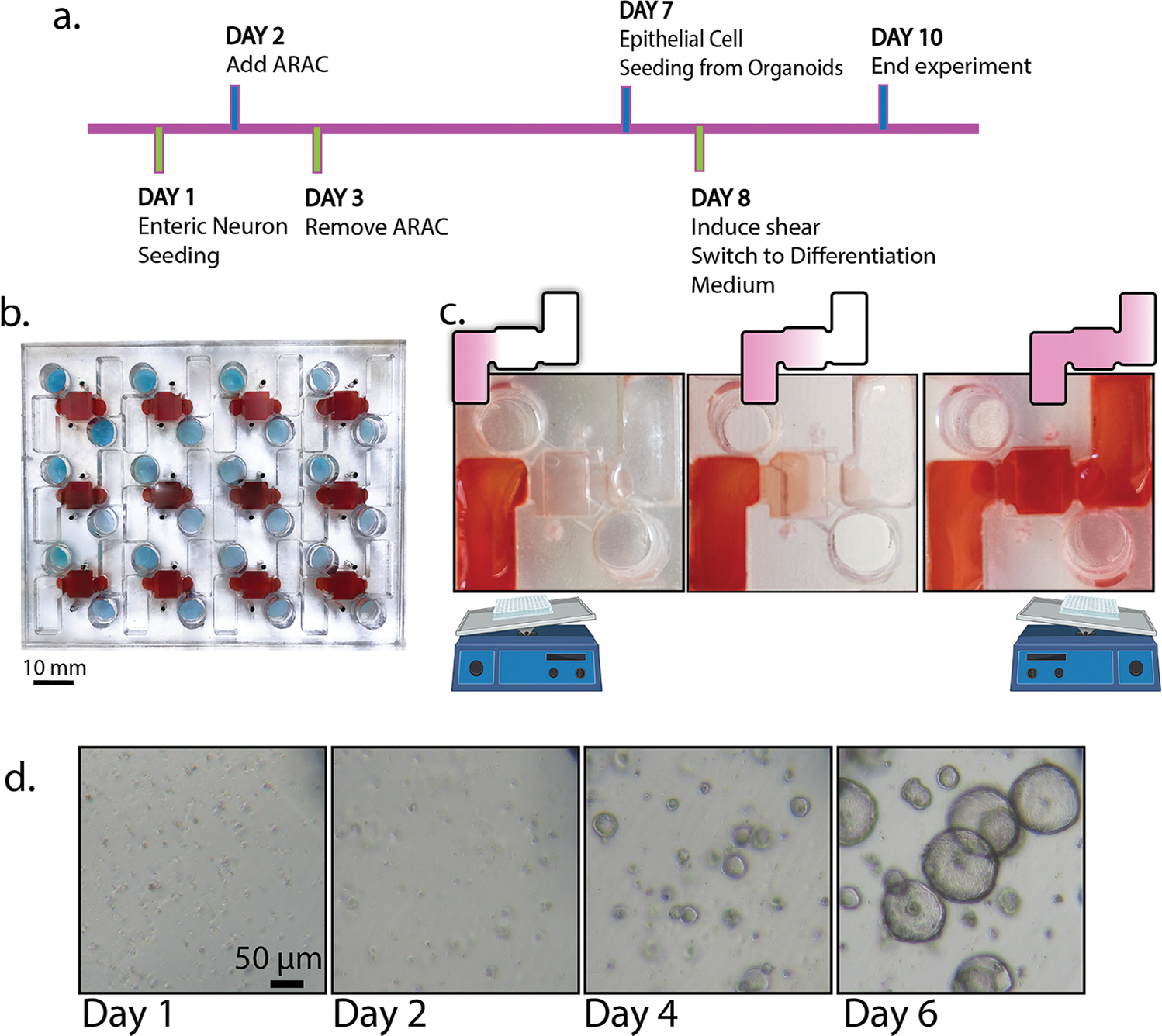
High-throughput, rocking chip design allows application of relevant shear stress without the need for tubing or pumps. a) Seeding timeline of the MPS with primary rat cells and the duration of experiments were run over 10 days, including 3 days in co-culture. Improvements to our model included b) scaling up the device to contain 12 independent culture systems (red marks a portion of the apical media channel including the culture area, blue denotes the basal media chamber) and c) supplying gravity-driven flow across the epithelial cells (red dye shows the flow through the entire apical media chamber). d) Epithelial crypt cells were isolated from the duodenum and expanded as intestinal organoids in a 3D culture environment before use in the experiments as shown in brightfield.

Optimization of the experimental timeframe was completed to support enteric neuron maturation and extension in the 3D environment of the MPS, as well as epithelial monolayer differentiation (Figure 2a). Neurons were allowed to mature for 7 days within the MPS before epithelial seeding to allow adequate time for neurite extension throughout the gel layer of the device. Epithelial cells were added to the MPS on the 7th day of neuronal culture. Neurons and epithelium were co-cultured for 3 days before endpoint analysis. Epithelial cultures were halted after 3 days in co-culture due to the reported high turnover rate of small intestinal epithelial cells, limiting the time frame of these experiments without a source for intestinal renewal.^[35,36]^ This epithelial shedding in vitro may be similar to anoikis in the gut where shedding of the intestinal lining occurs after 2–6 days.^[37]^ Twenty-four hours after epithelial seeding, the MPSs were moved from static culture conditions^[35,36]^ to a laboratory rocker to supply shear across the epithelial monolayer. A complete timeline of the experimental design is in Figure 2a.

To improve the robustness and ease of the MPS, the model was designed with two large, opposing media chambers to allow the application of relevant shear stress in a pump-free design (Figure 1c,d). Pumpless flow was incorporated into the device via rocking to make the model more biologically relevant than static cultures, as epithelial cells undergo shearing in the process of digestion and absorption.^[38,39]^ Flow of medium across the epithelial monolayer was accomplished with a standard platform rocker (Figure 2c). The large plate design eases alignment with the axis of rotation and ensures application of flow across the 12 MPSs. The flowing liquid induced by the rocker creates a more biomimetic environment for the intestinal cells than a static culture and avoids the use of bulky pumps and tubing that are often used in microfluidic devices to obtain shear stress.

Tilt angle and speed were adjusted to achieve pulsatile shear stress in the physiological range of 0.002–0.08 dyne cm^−1^.^[26]^ Our max shear stress was calculated to be 0.0683 dyne cm^−2^ across the middle of the cell culture chamber when the rocker was at a maximum tilt angle of 2 degrees and rocking at 10 rpm.


(1) $$\theta_{0}=2\left(\frac{{\;h}_{0}}{L}\right)$$

(2) $$\tau_{\omega }={\left.\mu \frac{\partial u}{\partial \varkappa }\right|}_{\varkappa =0}=\left\{\begin{array}{l} \frac{3\pi \mu \theta_{max}x(L-x)}{T{\left[{\;h}_{0}\;cot\;\theta +\frac{L}{2}-x\right]}^{2}{sin}^{2}\;\theta }cos\frac{2\pi t}{T},\theta \leq \theta_{0}\\ \frac{3\pi \mu \theta_{max}x}{T\left(\sqrt{2{\;h}_{0}L\;cot\;\theta }-x\right){sin}^{2}\;\theta }cos\frac{2\pi t}{T},\theta >\theta_{0} \end{array}\right.$$

Equation 1 was used to calculate the critical angle when the fluid-free surface comes in contact with the edge of the MPS bottom (16.7 degrees). Equation 2 was then used to calculate the max shear stress. Viscosity is a critical component of shear stress. Here, we used a published value of DMEM’s viscosity, 0.731 mPa s^−1^.^[40]^ The shear calculation used several assumptions, including a no-slip boundary condition at the bottom of the dish, a zero-velocity gradient at the fluid-free surface, flow mainly from gravity, and the effect from gravity is much greater than viscous effects. In addition, centrifugal force was neglected due to slow angular speed, velocity was assumed to be normal to the dish bottom, and the pressure gradient along the fluid depth was ignored.^[41]^ Once we calculated a shear value, we assumed our shear stress would remain the same across all experiments utilizing the noted parameters of the laboratory rocker.

Primary neonatal rat enteric neurons were cultured in three-dimensions in the MPS. Epithelial monolayers were seeded from rat neonatal duodenal organoids (Figure 2d). Using neonatal intestines allowed us to look at early epithelial development and differentiation. Most organoids maintained a spheroid morphology with limited budding up to seven days before passage. Seven days post neuron seeding, epithelial cells from organoids were seeded onto the top membrane layer of the chip to form a monolayer. Epithelial cells received a differentiation medium and were placed on the rocker to supply shear 24 h after being seeded on chip. On the tenth day from initial neuron loading, experiments were ended. Cells used in this model were sourced from neonatal rat cells, which are less potentially less indicative of mature, human adult tissue.^[42]^

### 2.2. Epithelial Cell Adhesion and Enteric Neurite Extensions Occur in an Interfacing Co-Culture MPS

**Figure 3.**
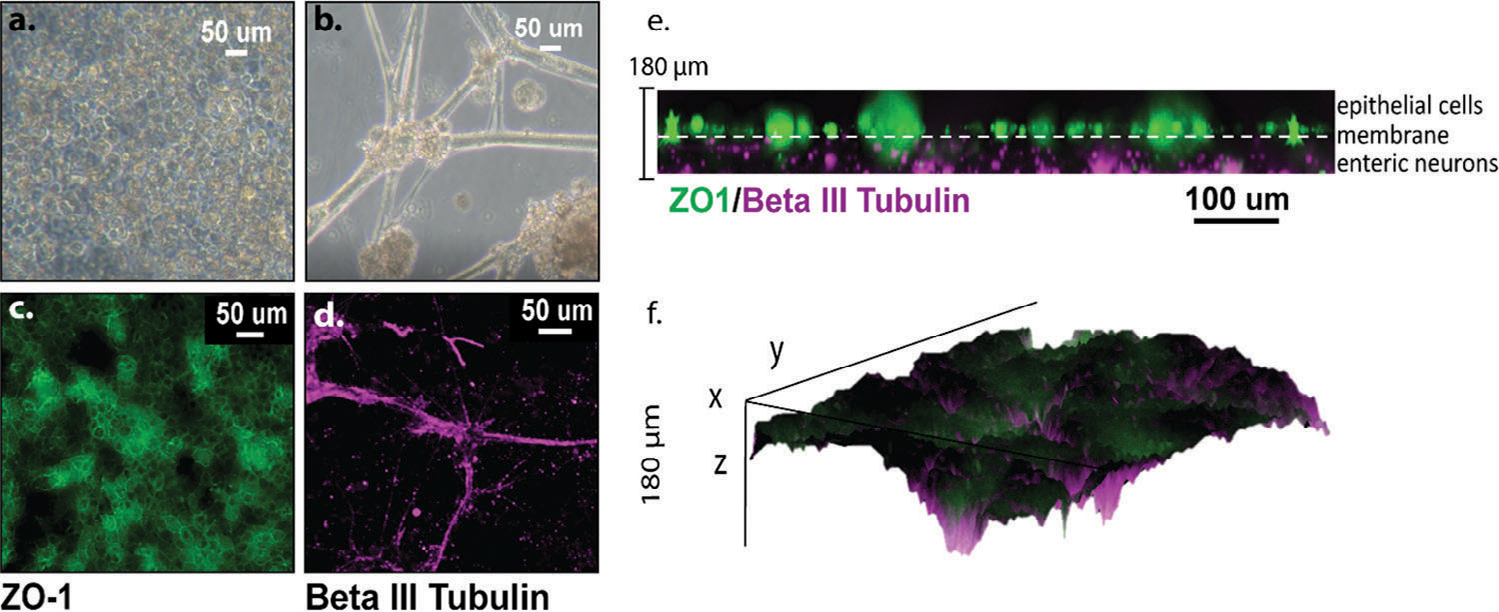
a) Representative brightfield images (day 10) of living epithelial and b) neuronal layers of the organ chip before fixing and staining. c) Immunostained layers of the MPS feature the epithelium, showing the ZO-1 tight junctions in the characteristic cobblestone pattern, and d) enteric neuron cultures stain with beta III tubulin. These images were taken in the same location at different focal planes in the z-direction. e) An orthogonal maximum intensity projection shows the layers of the culture, beta III tubulin identifying the neurons and ZO-1 the epithelium. f) A 3D surface plot showing the proximity of the different cell types in the culture systems and the depth.

The high throughput chip design was utilized for all cell culture experiments. Healthy cell morphologies with adherent epithelium and spindly neurons, were confirmed using an inverted light microscope prior to running endpoint experiments. The glass bottom and transparent membranes within the chip allowed for clear imaging through the different layers of the co-culture. Figure 3a,b shows the brightfield images of the epithelial monolayer on the top chamber and a plane of the gel containing the enteric neurons before fixation. The epithelial cells developed a cobblestone morphology adhering to the ECM-coated membrane, unlike the budding spheres they form while expanding. The enteric neurons developed neurite extensions, forming ganglionic-like morphology confirmed with imaging (Figure 3b,d). Mature epithelial cells were demonstrated by expression of ZO1 and a characteristic cobblestone pattern bordering the cells (Figure 3a,c). However, the cell surface did not maintain a uniform matured epithelium, exhibited by some areas of cytoplasmic ZO1 staining. This is likely due to enterocytes’ fast cell turnover period within the duodenum and 3-day timeframe for experiments. Figure 3d also features the neuron structural marker, beta III tubulin, within a single plane of the neuronal compartment of the MPS, highlighting the presence of enteric neurons in the co-culture device.

A unique feature of this system is that the 3D neuronal chamber is directly underneath, and proximal to, the epithelial monolayer. Transwell systems do not support the direct interfacing between cell populations we achieved in this MPS. The membrane pore size (1 μm) is large enough to support neurite extension through the gel layer and pores. This provides the potential of direct contact between the neurons and epithelium, facilitating both paracrine and synaptic communication. Orthogonal projections of a z-stack collected from our MPS show a distinct neuronal layer (purple, beta III tubulin) with some possible extensions through the membrane up to the top epithelial layer expressing ZO1 in green (Figure 3e). A 3D surface plot rendering of the ZO1 and beta III tubulin shows the intensities throughout the z stack of one of our co-cultured MPSs (Figure 3f). The surface plot displays the different depths that the cell populations reside in as well as the proximity of the neurons to the epithelium.

### 2.3. Enteric Neuron Cultures Represent a Heterogeneous Population with Cholinergic and VIPergic Subtypes Potentially Modulating the Epithelial Function

Enteric neurons interact closely with the gut epithelium in vivo but are traditionally absent from in vitro gut models. Much like the central nervous system, enteric neurons are composed of several subtypes responsible for different functions within the intestine, like modulating epithelial barrier formation and differentiation. We investigated the cholinergic and VIPergic neurons in our isolated cultures because of counteracting effects on the epithelium.^[26,28,29]^ Immunocytochemistry was utilized to assess the percentages of ChAT and VIP neurons present in our primary cultures (Figure S1a, Supporting Information). Our results showed a heterogeneous population of neurons with an average of 31.1% (±10.2%) expressing ChAT and 33.4% (±10.3%) expressing VIP (Figure S1b, Supporting Information). This was broken down further to quantify the ratios of ChAT only and VIP only expressing neurons, with 13.2% (±5.55%) expressing VIP only, 10.8% (±6.56%) expressing ChAT only, 20.3% (±7.82%) expressing both markers, and 53.8% (± 13.2%) expressing neither (Figure S1c,d, Supporting Information). Our findings differ slightly from prior literature estimating that the human small intestine contains 5–15% VIPergic and 50–70% cholinergic neurons.^[43–45]^ Differences could be due to the source organism and maturity, as the experiments presented in this work utilize neonatal rat tissue. The diversity of enteric neuron subtypes remains understudied in the literature. This brief analysis of our neuron cultures identified a majority VIPergic population, this subtype of neurons may contribute to a stronger barrier in the epithelial cells within our co-cultured system.^[26]^ However, several other neuronal subtypes have yet to be explored in this system and likely contribute to epithelial interactions.

### 2.4. Co-Cultured Epithelial Cells and Enteric Neurons had More Robust Barrier Properties Compared to Monocultured Epithelium

**Figure 4.**
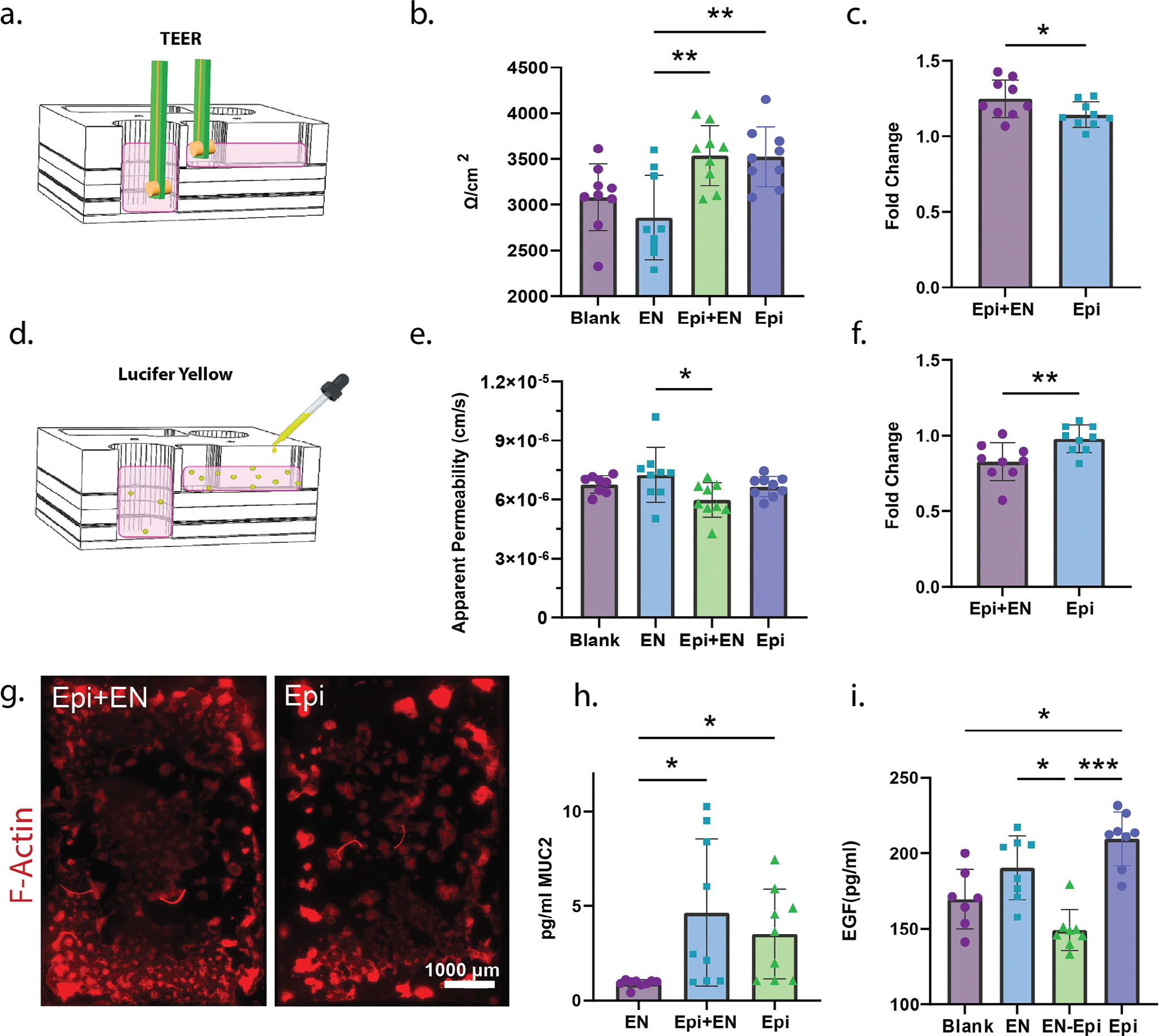
Our MPS system enables traditional barrier strength measures like transepithelial electrical resistance (TEER) and apparent permeability assay. a) TEER was measured via chopstick electrodes and the EVOM2. b) TEER values were overall greater for cultures containing the epithelium (Epi); *n* = 3, *m* = 3; error bars = SD; ** = *p* < 0.01, one-way ANOVA with multiple comparisons. c) Fold change of the TEER values controlled for the 3D gel layer degrading when ENS was cultured in it; *n* = 3, *m* = 3; error bars = SD; * = *p* < 0.05; two-tailed unpaired *t*-test. d) Apparent permeability, measured by lucifer yellow diffusion through the culture, followed the TEER results. e) Less fluorophore traveled through the layers when there was an epithelium present (n = 3, *m* = 3; error bars = SD; ** = *p* < 0.01; one-way ANOVA with multiple comparisons) and f) the fold change comparison to appropriate controls showed a significant difference between the epithelium with and without the ENS (n = 3, *m* = 3; error bars = SD; * = *p* < 0.05, *** = *p* < 0.001; two-tailed unpaired *t*-test). g) Cell area coverage was also measured using phalloidin-stained monolayers and images of the entire culture area. h) The concentration of Muc2 is higher in the epithelium containing cultures, as well as the co-culture show a trending increase in Muc2 production compared to the epithelium alone (n = 3, *m* = 2–3; error bars = SD; * = *p* < 0.05; one-way ANOVA with multiple comparisons). i) A higher EGF basal supernatant concentration was recorded from the co-culture condition than either of the monoculture conditions (n = 3, *m* = 2–3; error bars = SD; * = *p* < 0.05; *** = *p* < 0.001; Kruskal–Wallis test for non-parametric data).

Our 12-unit MPS system was designed to support real-time on-chip assays for assessing live epithelial barrier properties, including TEER and apparent permeability. TEER was measured using an EVOM device with chopstick electrodes on day 10 (Figure 4a). The design of the medium inlets was made large enough to support insertion of the electrodes into the chip, similar to a standard Transwell system (Figure 4a). On a single 12-unit chip, TEER values for the respective monoculture controls, epithelial and ENS co-cultures, and blank controls were measured and compared (Figure 4b). TEER values for enteric neuron only cultures were measured to use as an appropriate control against the co-cultured groups to account for changes the neurons might make to bulk resistance values. Enteric neuron-only control cultures exhibited lower resistances than the blank gel control (2860 ±462.6 Ω cm^−2^ versus 3083 ±365.3 Ω cm^−2^), likely because the glia degrade and migrate through the gel matrix, increasing porosity and resistance to passive ion transport.^[46]^ Although glia are reduced using ARaC, a fraction of glial cells persist and result in gel remodeling, likely through protease secretion.^[47]^ The fold change was calculated for each group, transforming the data by the appropriate control condition. The epithelial-only TEER values were transformed using the resistance from the blank gel, and the co-culture data was transformed using the enteric neuron resistance values. This fold change calculation controlled for resistance changes due to the neurons and allowed the assessment of the absolute TEER of the epithelial monolayers. There was a significantly higher fold change in the co-cultured group at 1.25 ±0.124 average fold, compared to the epithelium only at 1.14 ± 0.0835-fold (p = 0.049, Figure 4c).

Fluorescent molecule diffusion across the epithelium was measured in parallel to TEER on day 10. Here, we used the small fluorescent molecule Lucifer yellow to measure apparent permeability from the apical and basal compartments in our MPS over 3 h (Figure 4d). Figure 4e shows the raw apparent permeability values within our assay. The MPS thickness has a high ratio of media, membranes, and gel compared to the epithelial monolayer, resulting in insignificant changes to the raw apparent permeability values between co-cultured and monocultured groups. Fold change was similar to the TEER measurements, subtracting the permeability values of the two control groups (blank and neuron only) from the epithelial monoculture and co-cultured groups, respectively. There was a significantly lower permeability fold change of 0.83 ±0.125 for the epithelial and enteric neuron co-culture compared to the epithelial-only monolayer of 0.98 ±0.0904 fold (p = 0.0093, Figure 4f). These findings aligned with our TEER results and showed that enteric neurons may help boost epithelial integrity. These findings also highlight the ability to perform multiple high-throughput assays on-chip across several experimental groups.

In addition to permeability assays, we quantified the entire area of the MPS epithelial layer coverage by characterizing phalloidin-stained epithelial cells at the same timepoint as barrier function, on day 10 (Figure 4g). The edges of the tiled region are brighter due to reflection of the light from the acrylic. The area covered by cells for each sample was normalized by the average of all sample areas, combining mono- and co-culture, of a full technical replicate (n) to remove some interexperimental variability. The epithelial-only and co-cultured groups were compared, showing a higher normalized area coverage of 1.1048 ±0.291 for the co-cultured group with enteric neurons present compared to epithelial cells only with a mean normalized coverage of 0.8742 ±0.243 (Figure S2, Supporting Information). This supports our TEER and Lucifer yellow permeability findings and the hypothesis that enteric neurons promote an improved epithelium barrier function in our in vitro system. Enteric neurons have been found to increase cell proliferation and barrier integrity of the epithelium in previous experiments through tight junction and mucus formation.^[26,48,49]^ These past findings posed the question of if our microenvironments had detectable mucus concentrations.

### 2.5. Co-Cultures of Enteric Neurons and Epithelium Produce a Trending Increase in Mucus Compared to Epithelial Only Cultures

A Muc2 ELISA was performed with apical supernatants collected from epithelial cells cultured with and without neurons, as well as neuron only and blank media controls. Both the epithelial monoculture (2.43 ±1.50 pg mL^−1^, p = 0.048) and coculture (3.25 ±2.46 pg mL^−1^, p = 0.026) had significantly higher Muc2 concentrations than the enteric neuron monoculture (0.59 ±0.0796 pg mL^−1^), supporting mucus production in our MPS microenvironment (Figure 4h). Although co-cultures produced 33% more Muc2 than the monocultured epithelium, these findings were not statistically significant. From these results we cannot conclude that the barrier differences seen with neurons present were due to the increase in mucus in culture.

### 2.6. Co-Cultures of Enteric Neurons and Epithelium Contain Significantly Less EGF than Their Monoculture Counterparts

EGF is an important factor involved in regulating epithelial barrier proliferation and differentiation. An ELISA was run to measure the overall EGF concentration found in mono- and co-culture media supernatants due to the known impact on epithelial barrier function.^[50,51]^ The epithelial media contains 5 ng mL^−1^ of EGF added that either degrades or is consumed over the course of the experiment. The blank still contained 169.7 ±19.7 pg mL^−1^ which is significantly less than the monocultured epithelium (209.6 ±17.79 pg mL^−1^, p = 0.0013), suggesting the epithelium alone is producing EGF into the supernatant. The co-cultured epithelium and ENS contained significantly less EGF than either monoculture condition (Figure 4i). The mean EGF concentration of the co-cultured sample was 149.3 ±13.5 pg mL^−1^, compared to 190.3 ±21.05 and 209.6 ±17.79 pg mL^−1^ for neuron only and epithelium only, respectively (p = 0006 and p<0.0001). Our findings showed lower levels of EGF in the co-cultured epithelium supernatant which may represent a decrease in EGF production when neurons are present.^[50,52]^ The epithelium secretes EGF from enterocytes^[53]^ and Paneth cells,^[54]^ while the proliferative crypt cells have receptors for the EGF. The lower EGF in the supernatants could be due to fewer enterocytes or Paneth cells in the epithelium cultured with neurons, and potentially more intestinal epithelium stem cells. Further characterization of the composition of our epithelium under mono- and co-culture conditions would help us break down the source and consumption of EGF in our cultures. For the time being, the major takeaway is the enteric nervous system influences growth factor levels present in the intestinal epithelium and GI models lacking innervation are missing a vital component to gut renewal. This profound difference in EGF in the basal supernatant emphasizes the importance and great differences present in heterogeneous populations in in vitro models.

### 2.7. Wnt and Inflammatory Genes were Varied between the Monoculture and Co-Cultured Epithelium

**Figure 5.**
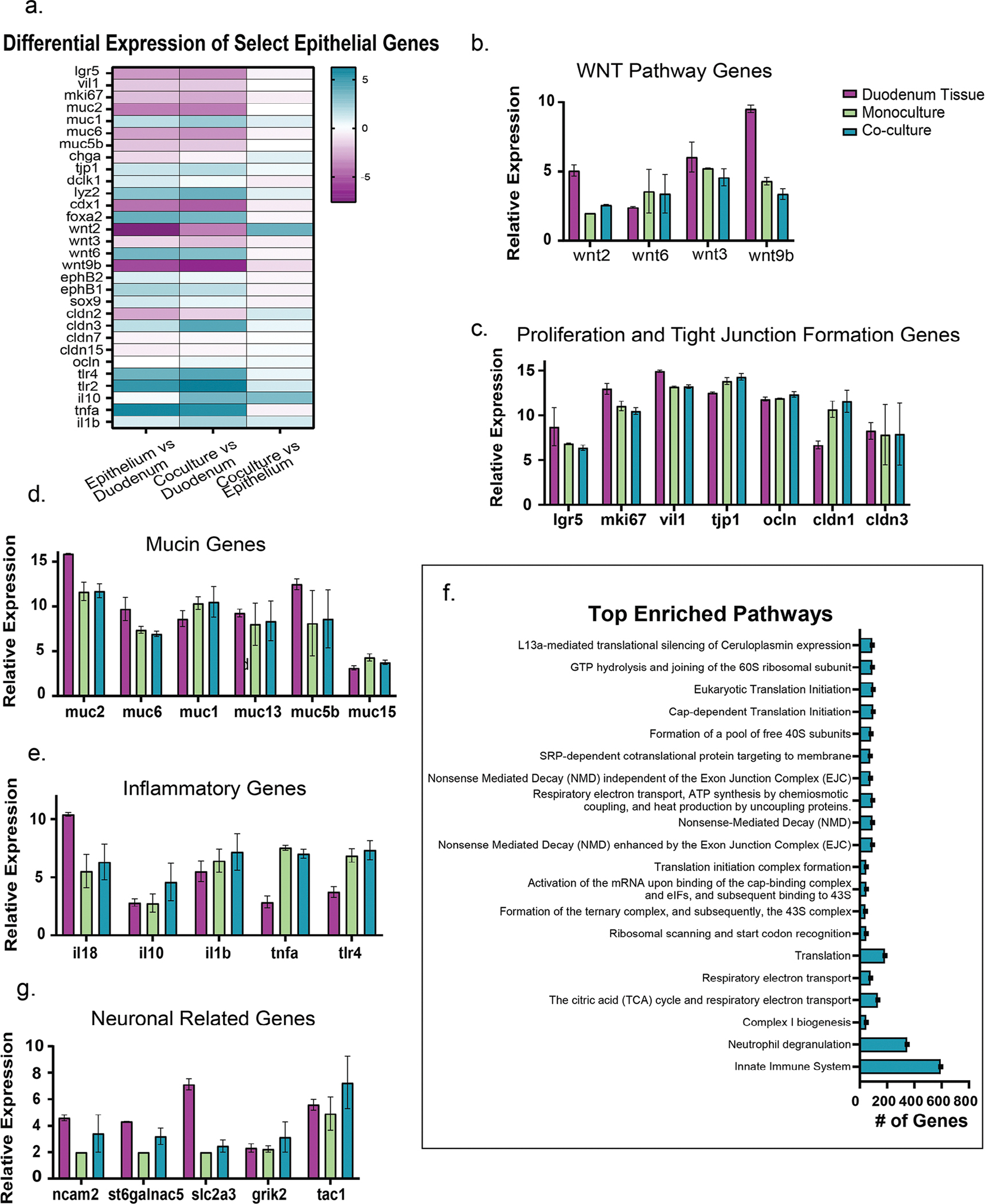
RNA sequencing analysis comparing freshly isolated duodenal crypts, MPS epithelium monoculture, and MPS epithelium in co-culture with enteric neurons. a) A heat map showing the differential gene expression, log2-fold change, of the experimental groups. Relative gene expression of categories of interest including: b) WNT, c) proliferation and tight junctions, d) mucus, and e) inflammation. f) The top 20 pathways enriched by the presence of neurons in our in vitro cultures. And g) neuron genes of interest. All analyses, n = 2 of 3 pooled samples per n, error bars = SEM.

Epithelial cells in monoculture and co-culture conditions were lifted from the MPS with TryplE. RNA sequencing was performed across two pooled samples for monocultured and co-cultured epithelium with 3 pooled monolayers per sample. Two samples of freshly isolated duodenal crypt tissue were also sequenced for comparison. Differential expression (log2-fold change) for epithelial genes of interest were compared across the three groups (Figure 5a). Relative expression levels for genes of interest were divided into functional categories (Wnt pathway, proliferation/barrier function, mucin production, and inflammatory cytokines) and reported (Figure 5b–e; Table S5, Supporting Information).

Wnt pathways related to Paneth cells (*Wnt3*, *Wnt6*, *Wnt9B*) had similar expression between the co-culture and monoculture.^[55]^ However, the co-culture group had a higher differential expression (3.74-fold, Figure 5a) of the *Wnt2* gene compared to epithelium monoculture. *Wnt2* has been found to inhibit inflammation and apoptosis in response to bacterial infection in vivo.^[56]^ And ablating and knocking down *Wnt2* function decreases the proliferation of colorectal cancer.^[57]^ Previous literature indicates that the *Wnt2* expression might be inhibiting apoptosis and increasing proliferation in our co-culture samples.

Genes associated with tight junction formation and proliferation were also compared (Figure 5c; Table S5, Supporting Information). Proliferation genes (*Lgr5*, *Mki67*) were slightly higher for monoculture samples than co-cultured (0.48- and 0.64-fold, respectively), but both lower than duodenal tissue. Tight junction protein genes were similar (*Tjp1*, *Vil1*, *Ocln*, *Cldn3*), except for claudin 1 (*Cldn1*) which had higher expression in the MPS samples than the tissue, with the co-culture group being the highest. A higher expression of *Cldn1* could indicate a population of more goblet cells and/or Paneth cells.^[58,59]^ Mucin gene expression was also explored in our work (Figure 5d; Table S5, Supporting Information), with most mucins being expressed in lower levels in the MPS groups compared to duodenal tissue (*Muc2*, *Muc6*, *Muc5b*), aside from *Muc1*. The media composition utilized in these experiments was aiming to maintain a monolayer for longer by enriching for proliferative cell types (including valproic acid and CHIR99021)^[60,61]^ and inhibiting apoptosis (Y-27632, ROCK inhibitor) which may have decreased the overall quantity of mucus producing goblet cells in these cultures as compared to the freshly isolated tissue. However, mucin producing genes were expressed similarly across the MPS samples, not matching the trend seen in the Muc2 production ELISA. Along with barrier formation specific genes, inflammatory genes were investigated as these can influence overall epithelial health (Figure 5e; Table S5, Supporting Information). Generally, the MPS samples showed higher levels of inflammatory markers like interleukins (*IL-10*, *IL-1b*) and toll like receptor genes (*Tlr4*, *Tlr2*) compared to the duodenal tissue. *Tnfa* and *Tlr4* had higher expression in the MPS samples compared to tissue. However, *IL-18* was lower in MPS samples compared to duodenal crypt tissue. The co-culture and monoculture samples were similar across most inflammatory genes of interest, except for *IL-10* which was 3.24-fold higher in the cocultured group. IL-10 has been found to have a number of effects on the intestinal epithelium including supporting differentiation into goblet and Paneth cells, supporting stem cell proliferation, promoting cell repair, and preventing apoptosis.^[62]^ Epithelial cells that interacted with neurons were found to have higher expression levels for the anti-inflammatory cytokine, IL-10, which matches previous data for non-contacting co-cultures in a Transwell system.^[63]^ Our work suggests that enteric neurons promote the expression of *IL-10* in duodenal epithelium. And the combined trends of enriched *Wnt*, *Cldn1*, and *IL-10* genes might suggest our co-cultured samples contain more Paneth cells or goblet cells than the monocultured epithelium. However, the Paneth cell (*Lyz2*) and goblet cell (*Muc* group of genes) are not as profoundly enriched to support this conclusion.^[64]^

Figure 5e lists the top 20 pathways effected by the presence or absence of ENS on the epithelium in MPS based on normalized enrichment scores (NES, scores reported in Table S6 (Supporting Information) gene counts listed in Figure 5e). Interestingly, innate immunity and neutrophil degranulation had the highest number of gene hits. The pathway with the highest enrichment score was L13a-mediated translational silencing of ceruloplasmin expression which is also contributes to immune function.^[65]^

### 2.8. Epithelial RNA from Co-Cultured MPSs have Increased Neuronal Gene Expression Suggesting Presence of Extending Neurites to the Epithelial Monolayer

The top 100 differential genes were compared for the monocultured and co-cultured MPS samples (Table S7, Supporting Information). Within these top genes, several were found to be prevalently expressed within axons and dendrites, including *Ncam2* which was the top differentially expressed gene (6.29-fold in co-culture versus monoculture, Figure 5g). Other top hits of neuronal genes included *St6galnac5*, *Slc2a3*, *Grik2*, and *Tac1*.

The effect of the neurons may have been from direct contact between the neurites and the epithelium. Some of the top differential genes were related to neuronal function and found in the neuron extensions, like *Ncam2*. This supports our hypothesis that neurites are extending through our porous membrane (1 μm pore size) into the epithelium culture, and when mRNA was collected neural fragments were lifted from the top membrane as well. Relative gene expression analysis of acetylcholine and VIP related receptor genes can be found in Figure S3 (Supporting Information).

Several of the top twenty enriched pathways (Figure 5f) were related to immune response. However, our system lacks the inclusion of specific immune cells. Adding complexity to the MPS by incorporating mast or dendritic cells may further advance the platform’s ability to maintain homeostasis and closer match what would be seen in the body. Some key genes related to cytokine production or inflammatory activation differed between the MPS and duodenal crypt samples, including *Tnfa* and *Tlr4* gene expression. These genes were expressed at higher levels in both of the MPS samples compared to tissue. This may be due to the epithelium being heavily differentiated and undergoing anoikis when cultured in vitro. Anoikis, a programmed cell death that enterocytes undergo within the intestinal lining, involves TNF-*α* reception with tumor necrosis factor receptor 1 (Tnfr1), often termed a death receptor due to the presence of a death domain.^[66]^ We hypothesize in vitro monolayers contain a greater ratio of cells undergoing anoikis compared to in vivo, resulting in heightened levels of *Tnfa* expression. A model that incorporates a self-renewing monolayer with a higher ratio of Lgr5 positive cells present may decrease the differential expression seen between MPS and tissue samples. Along with *Tnfa*, *Tlr4* is implicated in pro-inflammatory response and anoikis via the NF-ΚΒ signaling pathway. This pathway promotes the transcription of proinflammatory genes, including those for *Il1b* and *Tnfa*. NF-ΚΒ is also involved in a cascade related to epithelial-mesenchymal transition (EMT) which can be activated via TLR4 and TNF-*α* signaling and causes epithelial cells to depolarize and detach from the extracellular matrix.^[67,68]^ The cells can then migrate and proliferate as needed to other sites. EMT is an important process related to stem cell maintenance, inflammation, and injury repair and could be activated in our system’s monolayers due to the conditions of the microenvironment through factors such as cytokine production, altered WNT pathways, and growth factors.^[69,70]^ For example, we saw decreased EGF levels in our co-culture system via ELISA and increased *Wnt2* expression, which may alter EMT when neurons are present, and therefore improve the epithelial barrier integrity. All of this is speculation that could be further explored in future studies that include more in-depth quantification of cytokines and growth factors present in the system, as well as the incorporation of immune cells. Incorporating the complex and branching architectures of the vascular and lymphatic systems may further influence cytokine expression and transport phenomena.^[71]^

Modeling the duodenum was chosen since it is a heavily innervated part of the intestine and where the bulk of drug absorption occurs,^[72]^ and it is underrepresented in current literature. However, duodenal organoids and monolayers are not as robust or well documented in literature as those from the colon.^[73–75]^ Colonic tissue has a slower turnover rate and higher levels of mucus producing goblet cells. The slower turnover rate of colonic cells translates to longer-lasting monolayer viability compared to duodenal cells that will differentiate quickly and undergo apoptosis. The increased goblet cells, and as a result mucus barrier, of the colon also provides a more substantial quantifiable barrier. Incorporating enteric neurons may be a valuable method for improving the quality of in vitro duodenal cultures, expanding the ability to study small intestine disorders.

## 3. Conclusion

Through the work presented herein, a new small intestine organ-on-a-chip was designed, validated, and characterized. Design improvements to our intestinal microphysiological system include increased throughput, gravity driven flow, focused on duodenal tissue, and the added complexity of the enteric nervous system. Our results display that enteric neurons influence the function and health of the intestinal epithelium without an exogenous or inflammatory challenge. Cultures including the enteric nervous system and intestinal epithelium had significantly stronger barrier function, changes in extracellular EGF, and increases in RNA encoding for cytokine and innate immunity genes. Given the poorly understood, and consequently insufficient treatments, for GI disorders like IBD and IBS; improving our understanding of cellular interactions at the tissue level could improve drug development to target these disorders. This work proves that future steps should strive to include more complex tissues in GI models like the ENS. The model established in this work is the first innervated, small intestinal MPS. This work is a jumping-off point for future studies of neuronal communication to and from the gut.

## 4. Experimental Section

### MPS Fabrication:

Innervated MPSs were assembled layer-by-layer using laser cut thermoplastics with our established method, however we scaled the system to include twelve individual “chips” onto one device.^[8,32]^ Transparent, cast polymethyl methacrylate (PMMA, *McMaster-Carr, 8560K211* and *8560K171*) and clear 0.005-inch polyethylene terephthalate (PET) film (*McMaster-Carr, 8567K54*) were used for the cell chamber wall layers. Tissue culture-treated PET, semi-permeable membranes with 1 um pores (*It4Ip, ipCellCulture 2000M12/620M103*) were utilized to separate the cell populations. Layered assembly was done using pressure-sensitive transfer tape (*3M, 966*), and the chip was built on top of a glass slide base layer (*Ted Pella, 260230–50*). Designs for each MPS layer were created as vectors in Adobe Illustrator and cut using an Epilog Zing laser cutter. Transfer tape was laminated onto the desired layers before laser cutting. MPSs were assembled by hand, heat pressed, and then placed into a vacuum oven for at least 120 h at 50°C to allow curing and off-gassing of the adhesive material. Binder clips were used to apply additional pressure to the newly assembled MPS while baking.

### Animal Care and Use:

The following tissue isolation procedures were approved by Northeastern University’s Institutional Animal Care and Use Committee (IACUC) under protocol 23–0305R. All methods were performed following the guidelines and regulations necessary.

### Neuron Isolation and Cell Culture:

Enteric neurons were isolated from neonatal, two-day-old Sprague-Dawley rats (p2) sacrificed via decapitation (*Charles River, Crl:CD(SD); RRID: RGD_734476*). Rat pups were mixed sex. The small intestines were removed, and the myenteric plexus was carefully peeled off the intestines using forceps under a dissecting microscope and stored in ice-cold Hanks Balanced Salt Solution (HBSS). The peeled myenteric plexus was moved into a 15 mL conical tube and digested for 1 h in collagenase type II (*Gibco, 17101015*, 1 mg mL^−1^) and Deoxyribonuclease I (*Sigma*, 0.5 mg ml^−1^) dissolved in neurobasal medium (*Gibco, 10888022*) at 37°C. The myenteric plexus in the digestion medium was vortexed for 5 s and examined visually. The digestion continued in 30-min increments until the myenteric plexus was visually broken up into small particles. Typically, this is complete after three additional 30-min digestion steps. The solution was then centrifuged at 500 g for 5 min, and the supernatant was removed. The pellet was resuspended in a 0.05% trypsin-EDTA (*Gibco, 25200056*) solution for 30 min at 37° C to dissociate the neurons. After dissociation, the solution was centrifuged at 500 g for 5 min, and the trypsin supernatant was removed. The cell pellet was resuspended in enteric neuron medium (Table S1, Supporting Information) and counted for culture. The fully digested neural tissue was seeded directly onto tissue culture treated glass, nonadherent well plates, or directly into the MPS (methods for gel encapsulation and seeding described later). Neurospheres were kept in suspension culture with enteric neuron media without the addition of any growth factors (NGF and GDNF), adherent or 3D cultures were fed the complete media as described in Table S1 (Supporting Information). Media was refreshed every 2–3 days either by half exchanging media every two to three days in adherent and 3D conditions or fresh media was added on top of the suspension cultures.

### Epithelial Organoid Isolation:

Duodenal epithelial organoids were isolated from the first 3–4 cm of the small intestine of neonatal rat pups (p2). After the myenteric plexus was removed from the segment, the remaining intestine was fileted open and cut into small pieces (≈5 mm). The diced intestines were washed ten times with cold Hanks balanced salt solution (HBSS). With the final wash, the HBSS was removed and replaced with 2 mM EDTA (*Invitrogen, AM9260*) in HBSS. The tissue was left to digest on ice and rocking for 15 min. Then the tissue was allowed to settle, and the EDTA solution was removed and replaced with fresh 2 mM EDTA and digested for an additional 25 min on ice with rocking. When digestion was complete, crypts were detached from the rest of the tissue by vortexing the tube for 1 min, followed by pipetting vigorously using a 10 mL serological pipette while mixing about 25 times. The suspension was filtered through a 100 μm cell strainer and spun down at 300g for 5 min. The supernatant was removed, and the pelleted crypts were resuspended in Advanced DMEM/F12 (*Gibco, 12634028*) and spun again to wash, this was repeated three times. After the final wash and suspension removal, 20 μL of crypts were encapsulated in 1 mL growth factor reduced Matrigel (*Corning, 356231*) and then plated into 50 μL domes in a 12-well plate, three domes per well. After the Matrigel had polymerized, the cells were fed an expansion medium (Table S2, Supporting Information) with an addition of 10 μM rock inhibitor for the first feed after isolation or replating, Y-27632. Organoids were fed every 2–3 days with fresh expansion medium and passaged every 5–7 days.

Organoids were passaged by removing the media, replacing it with Cell Recovery Solution (*Corning, 354270*), and then disrupting the Matrigel domes by scraping them off the plate with a pipette tip. Organoids, Matrigel fragments, and cell recovery solution were collected into a tube and left on ice rocking for 45 min to degrade the Matrigel. Organoids were then spun down at 500 g for 5 min, and the supernatant was removed. At this point, 20 μL of the pellet was collected and resuspended in 1 mL of Matrigel, or the cells underwent further digestion for monolayer seeding.

### MPS Seeding Protocol:

Fabricated co-culture chips were sterilized by UV for 10 min, followed by oxygen plasma treatment for 90 s to prepare the membrane for protein coatings. The top membrane was coated with poly-d-lysine (*Gibco, A3890401*) and incubated for 1 h at 37° C, and then rinsed with PBS at least twice. Next, a 1:10 dilution of Matrigel coating was added on top of the poly-d-lysine in preparation for epithelial monolayer adhesion. The MPS and Matrigel was incubated for at least 1 h at 37° C before chips were seeded with the hydrogel and enteric neurons.

To seed organ chips with the neurospheres, the enteric neuron media with suspended neurospheres was first collected from the wells using a 1000 μL pipette and placed into a 15 mL tube. The tube was centrifuged at 300g for 5 min to pellet the neurospheres. The supernatant was removed, and 1 mL of Accutase (*Corning, 25058CI*) was added to the pellet. The Accutase solution was triturated to break up the pellet, and the tube was incubated for 30 min at 37° C. After incubation, the neurospheres were triturated 25–45 times using a 1 mL pipette tip and centrifuged at 300g for 5 min. The Accutase was removed and replaced with fresh neuron medium, and cells were counted and seeded on-chip at 1 × 10^6^ cells mL^−1^ in a 10% Matrigel (*Corning, 356231*), 2 mg mL^−1^ Cultrex Collagen (*R&D Systems, 344702001*) solution in neuron media. The volume of cells needed to achieve 1 × 10^6^ cells mL^−1^ was mixed with the Matrigel and Cultrex solution and 10 μL was injected into inlets on the MPS that lead into the enteric neuron chamber. The gels were thermally crosslinked at 37°C for 20 min before medium was added to the reservoirs of the chip. Neurons received a complete media exchange after 24 h with 20 μM of the mitotic inhibitor, cytosine *β*-D-arabinofuranoside (AraC, *Sigma, C1768*). During the remaining timeframe, the neurons received half-volume media exchanges.

After a week of neuron culture maturation, we seeded the epithelial monolayer. The Matrigel domes were disrupted with manual scraping using a pipette tip. Organoids and Matrigel fragments were collected in cell recovery solution and rocked on ice for 45 min to remove the organoids from the Matrigel. Organoids were centrifuged at 500g for 5 min, and the cell recovery solution was removed. Organoids were resuspended in trypLE (*Gibco, 12563*) and digested for 5 min at 37° C. The cells were then agitated by pipetting up and down through a bent 1 mL pipette tip 20 times to achieve a single-cell suspension. Cells were spun down to remove the trypLE, resuspended in the expansion medium with 10uM Y-27632 rock inhibitor, and counted. Chips were seeded with 600,000 cell per chip, 2.2 × 10^6^ cells cm^−2^. After one day, the media was changed out for differentiation medium (Table S3, Supporting Information).

### Media Compositions:

According to supplier suggestions, lyophilized reagents were resuspended to their working concentration in deionized water, DMSO, or PBS. Rat enteric neuron medium was used for feeding neuron cultures (Table S1, Supporting Information), with a half volume exchange performed every two to three days. Rat organoid proliferation medium (Table S2, Supporting Information) was used during organoid expansion in Matrigel domes and for the first 24 h of seeding a monolayer. For chip monolayer experiments, after 24 h, the epithelial medium reservoir was exchanged for rat organoid differentiation medium (Table S3, Supporting Information) to encourage differentiation into representative heterogeneous cell populations.

### Application of Shear:

MPSs were placed on a platform rocker (*Thermo-Scientific, 11–676-680*) in an incubator with 5% CO_2_ at 37° C for three days. A rocker tilt angle of 2° and rotation speed of 10 rpm were determined to generate a physiological rate of shear force (0.002–0.08 dyne cm^−2^), calculated at the center of the monolayer culture surface area when at the maximum tilt angle^6^. Calculations were determined using the assumptions and formulas stated in a previous publication.^[41]^ The critical flip angle, *θ*_0_, when the fluid-free surface first contacts the edge of the bottom of the MPS, was calculated using the MPS’s epithelial channel dimensions (Equation 1). Here, *h*_*0*_ represents the medium depth, and L represents the length of the epithelial channel. Once the critical flip angle was determined, the shear stress could be calculated using Equation 2, where *θ* is the rocking angle, *θ*_*0*_ is the critical flip angle, *T* is the period, and *x* is the location of interest within the channel.

### Lucifer Yellow Permeability Assay:

Lucifer yellow lithium salt (*Invitrogen, L453*) was resuspended to a 100 μM stock solution in PBS and wrapped in foil to avoid light exposure. A 10 mM working solution was prepared in phenol red free DMEM (*Gibco, 31053028*). All media was carefully removed from apical and basal chip compartments, and 150 μL of 10 mM lucifer yellow was added into the apical compartments of all the chip wells. Phenol red free DMEM was added to the basal compartments (150 μL) as well. The chips were incubated at 37°C for 3 h at static conditions. After incubation, 100 μL of the solution was removed from the apical and basal channels and put into separate wells of a black 96-well plate. A standard curve for the lucifer yellow working solution was prepared using 1:2 serial dilutions of the 10 mM solution and phenol red free DMEM. The fluorescence was then measured for each well on a plate reader spectrophotometer using 428/530 excitation/emission settings with the gain set between 50 and 75. Apparent permeability was then calculated using the concentration values determined from a linear fit of the standard curve.

### Immunostaining:

Cells were fixed with paraformaldehyde for 10 min. After fixation, the solution was removed from the culture chambers, and a rinsed with HBSS twice. Cells were permeabilized in a 0.1% Tween-20 solution for 10 min. After rinsing out the Tween, a blocking solution of filtered 4% goat serum (*Sigma, G9023*) was added to the culture and left to incubate overnight at 4° C. The following day, the blocking solution was removed. Antibodies (Table S4, Supporting Information) diluted in 4% goat serum were added into the wells and incubated for 2 h at room temperature or overnight at 4° C. Following antibody addition, MPS compartments were carefully rinsed with HBSS three times. Secondary antibodies diluted in 4% goat serum were then added and incubated for 2 h at room temperature or overnight at 4° C. Following secondary incubation, DAPI (*Invitrogen, D1306*) was added at 1:1000 dilution in PBS for 10 min. Rinsing was then performed carefully in the MPS at least three times. MPSs were imaged immediately or stored at 4° C and wrapped with parafilm.

### Terminal Epithelial Monolayer Characterization:

Monolayers were stained for filamentous actin with Phalloidin to identify the cell cytoskeleton and total area. Tiled images of the entire culture area of the immunostained MPSs were acquired on a Zeiss Axio Observer Z1 (*Carl Zeiss Microscopy*). Exported images were analyzed using Fiji in ImageJ.^[76]^ Briefly, the images were cropped to a uniform size matching the dimensions of the chip culture area. Then a variance filter was applied to improve contrast at the edges of the cells. The MorphoLibJ plugin was used to segment the image, and the total cell area covered was recorded.^[77]^

### Acetylcholine and Vasoactive Intestinal Peptide Neuron Subtype Quantification:

Neonatal rat enteric neurons were isolated as described above and plated on cover glasses. Cover glasses were sterilized by UV for 5 min on each side, oxygen plasma treated for 90 s, poly-d-lysine (*Gibco, A3890401)* coated for 1 h followed by washes, and finally 50 μg mL^−1^ mouse laminin (*Corning, 354232*) coating for 30 min. Neurons were seeded at a density of 50 000 cells per well in a 24-well plate with a half-exchange medium feeding every two to three days. After 1 week, the neurons were fixed with 4% paraformaldehyde for 10 min and then washed with HBSS 3 times. The cells were permeabilized with 0.1% Triton-X for 10 min, followed by additional washes with HBBS. Blocking was done with 2.5% goat serum (*Sigma, G9023*) for at least 1 h before adding the primary antibodies for Beta III Tubulin, VIP, and ChAT (see product numbers and dilutions in Table S4, Supporting Information). Primary antibodies were incubated at room temperature for at least 1 h, followed by 3 washes with HBSS. The secondary antibody solution was similarly made in 2.5% goat serum and incubated at room temperature for at least 1 h, followed by 3 washes. Cover glasses were then mounted on microscope slides with ProLong Gold Antifade Mountant and DNA Stain DAPI (*Invitrogen, P36931*).

Images of the stained neurons were segmented and quantified using Cell Profiler.^[78]^ Regions of interest were segmented using the nuclei stain of DAPI as a seed and Beta III Tubulin for the boundaries of the cell. Individual cells were quantified for their integrated intensity, the sum of pixel intensities per region of interest. They were considered positively stained for VIP or ChAT if they were greater than 1 standard deviation from the mean of all the neurons imaged per experimental replicate. Immunostained samples were imaged on a Zeiss Axio Observer Z1 (*Carl Zeiss Microscopy*) for the MPS z-stacks, MPS orthogonal projections, neuronal subtype and phalloidin cell coverage experiments.

### Transepithelial Electrical Resistance (TEER) Measurements:

TEER was performed using a World Precision Instruments EVOM device with chopstick electrodes. Electrodes were first cleaned in 70% ethanol and calibrated in PBS. Recordings were made by completely submerging the electrodes into the media of the apical and basal chambers and allowing the resistance reading to equilibrate. A recording was taken for each basal inlet of each sample and averaged. TEER calculations were then normalized to the area of the chip (0.49 cm^2^) to get Ω cm^−2^.

### ELISA Assays: EGF and Muc2 Secretion:

At experiment end points, cell culture supernatants were collected separately from each sample’s apical and basal compartments and centrifuged at 3000 rpm for 10 min at 4°C. The centrifuged supernatants were moved into a clean microcentrifuge tube and stored immediately at −80°C until needed. An EGF ELISA (*Invitrogen, EREGF*) was performed on the collected basal supernatants following supplier instructions with samples ran in duplicate and averaged. A Muc2 ELISA (*Novus Biologicals*, NBP276701) was performed similarly using the apical supernatants. Optical densities were obtained using a plate reader at 620 nm and compared to a standard curve fit.

### RNA Sequencing Methods:

Epithelial cells monocultured and co-cultured in the MPS devices were lifted from the membrane using 0.25% trypsin-EDTA *(Gibco, 25200056)*. Cells were incubated in trypsin-EDTA for 10-min increments and agitated via pipetting to release them from the membrane. The culture area was checked under the microscope after each 10-min incubation to ensure the majority of the cells had been lifted. Cells were then pipetted into a microcentrifuge tube and centrifuged at 500g for 5 min to form a pellet. The cell pellets were rinsed with HBSS and spun again. HBSS was removed and cell pellets were stored dry at −80°C until shipment. Three samples of epithelial cells for each condition (monoculture and co-culture with neurons) were pooled into two independent samples. Pooling allowed adequate collection of RNA since MPS chamber volumes are small. Samples were sent overnight on dry ice to Azenta Life Sciences (New Jersey) for next generation sequencing (NGS) services. RNA was extracted by Azenta and appropriate quality checks were performed. Low input RNA sequencing methodology was utilized using an Illumina System and the raw counts with gene IDs were provided by the supplier. Differential and relative gene expression, as well as top enriched pathways were quantified using the raw count files imported into iDEP.96.^[79]^ A log2-fold change method was used for differential expression and normalized enrichment scores (NES) and Reactome pathways were used for pathway enrichment analysis.^[80]^

### Statistical Analyses:

GraphPad Prism (v. 9.3.1) was used to run statistical testing. Normality tests were performed on each data set. Data sets with more than two experimental trials were analyzed for statistical significance (p < 0.05) using an unpaired *t*-test or one-way ANOVA with multiple comparisons for normally distributed data sets and a Kruskal–Wallis nonparametric test for non-normal data sets. All experiments, excluding the phalloidin area coverage and RNA sequencing, were performed independently in triplicate. For experiments using primary cells, litters of 10 mixed-sex rat pups were used and pooled per experimental trial.

## Supplementary Material

Supp Material

Supporting Information

Supporting Information is available from the Wiley Online Library or from the author.

## Data Availability

The data that support the findings of this study are available from the corresponding author upon reasonable request.

## References

[1] Hanning N, Edwinson AL, Ceuleers H, Peters SA, De Man JG, Hassett LC, De Winter BY, Grover M, Therap. Adv. Gastroenterol. 2021, 14, 175628482199358. 10.1177/1756284821993586 PMC7925957 33717210

[2] Michielan A, D’Inca R, Mediators Inflamm. 2015, 2015, 628157. 26582965 10.1155/2015/628157 PMC4637104

[3] Ohlsson L, Gustafsson A, Lavant E, Suneson K, Brundin L, Westrin Å, Ljunggren L, Lindqvist D, Acta Psychiatr. Scand. 2019, 139, 185. 30347427 10.1111/acps.12978 PMC6587489

[4] Kim S, Goel R, Kumar A, Qi Y, Lobaton G, Hosaka K, Mohammed M, Handberg E-M, Richards E-M, Pepine C-J, Raizada M-K, Clin. Sci. 2018, 132, 701. 10.1042/CS20180087 PMC5955695 29507058

[5] Beaurivage C, Kanapeckaite A, Loomans C, Erdmann KS, Stallen J, Janssen RA, Sci. Rep. 2020, 10, 1. 33293676 10.1038/s41598-020-78359-2 PMC7722760

[6] Kim HJ, Huh D, Hamilton G, Ingber DE, Lab Chip 2012, 12, 2165. 22434367 10.1039/c2lc40074j

[7] Poceviciute R, Ismagilov RF, Nat. Biomed. Eng. 2019, 3, 500. 31278388 10.1038/s41551-019-0425-0

[8] Hosic S, Bindas A, Puzan M, Lake W, Soucy JR, Zhou F, Koppes RA, Breault DT, Murthy SK, Koppes AN, ACS Biomater. Sci. Eng. 2020, 7, 2949. 34275297 10.1021/acsbiomaterials.0c00190 PMC8290094

[9] Lee SH, Sung JH, in Organ-On-A-chip, Elsevier, Amsterdam 2020, pp. 295–310.

[10] Zhang S, Xu G, Wu J, Liu X, Fan Y, Chen J, Wallace G, Gu Q, Small Methods 2024, 8, 2300685. 10.1002/smtd.202300685 37798902

[11] Tsamandouras N, Chen WLK, Edington CD, Stokes CL, Griffith LG, Cirit M, AAPS J 2017, 19, 1499. 10.1208/s12248-017-0122-4 PMC6540747 28752430

[12] Shin MK, Kim SK, Jung H, Lab Chip 2011, 11, 3880. 21975823 10.1039/c1lc20671k

[13] Lindner M, Laporte A, Block S, Elomaa L, Weinhart M, Cells 2021, 10, 2062. 34440830 10.3390/cells10082062 PMC8391940

[14] van der Helm MW, Odijk M, Frimat J-P, van der Meer AD, Eijkel JC, van den Berg A, Segerink LI, JoVE 2017, 127, 56334. 10.3791/56334 PMC5752338 28994800

[15] Henry OY, Villenave R, Cronce MJ, Leineweber WD, Benz MA, Ingber DE, LabChip. 2017, 17, 2264. 10.1039/c7lc00155j PMC5526048 28598479

[16] Yu LC-H, Wang J-T, Wei S-C, Ni Y-H, World J Gastrointest. Pathophysiol. 2012, 3, 27. 10.4291/wjgp.v3.i1.27 PMC3284523 22368784

[17] Tan H-Y, Trier S, Rahbek UL, Dufva M, Kutter JP, Andresen TL, PLoS One 2018, 13, 0197101. 10.1371/journal.pone.0197101 PMC5944968 29746551

[18] Rahman S, Ghiboub M, Donkers JM, van de Steeg E, van Tol EAF, Hakvoort TBM, de Jonge WJ, Int. J. Mol. Sci. 2021, 22, 13472. 34948271 10.3390/ijms222413472 PMC8709104

[19] Hedrich WD, Panzica-Kelly JM, Chen S-J, Strassle B, Hasson C, Lecureux L, Wang L, Chen W, Sherry T, Gan J, Davis M, Toxicology 2020, 446, 152614. 33199268 10.1016/j.tox.2020.152614

[20] Sugimoto S, Sato T, in 3D Cell Culture: Methods and Protocols, Springer, New York, 2017, pp. 97–105.

[21] Workman MJ, Mahe MM, Trisno S, Poling HM, Watson CL, Sundaram N, Chang C, Schiesser J, Aubert P, Stanley EG, Elefanty AG, Miyaoka Y, Mandegar MA, Conklin BR, Neunlist M, Brugmann SA, Helmrath MA, Wells JM, Nat. Med. 2017, 23, 49. 27869805 10.1038/nm.4233 PMC5562951

[22] Jarret A, Jackson R, Duizer C, Healy ME, Zhao J, Rone JM, Bielecki P, Sefik E, Roulis M, Rice T, Sivanathan KN, Zhou T, Solis AG, Honcharova-Biletska H, Vélez K, Hartner S, Low JS, Qu R, de Zoete MR, Palm NW, Ring AM, Weber A, Moor AE, Kluger Y, Nowarski R, Flavell RA, Cell 2020, 180, 50. 31923399 10.1016/j.cell.2019.12.016 PMC7339937

[23] Yan Y, Ramanan D, Rozenberg M, McGovern K, Rastelli D, Vijaykumar B, Yaghi O, Voisin T, Mosaheb M, Chiu I, Itzkovitz S, Rao M, Mathis D, Benoist C, Immunity 2021, 54, 499. 33691135 10.1016/j.immuni.2021.02.002 PMC8133394

[24] Drokhlyansky E, Smillie CS, Van Wittenberghe N, Ericsson M, Griffin GK, Eraslan G, Dionne D, Cuoco MS, Goder-Reiser MN, Sharova T, Kuksenko O, Aguirre AJ, Boland GM, Graham D, Rozenblatt-Rosen O, Xavier RJ, Regev A, Cell 2020, 182, 1606. 32888429 10.1016/j.cell.2020.08.003 PMC8358727

[25] Walsh KT, Zemper AE, Cell Mol. Gastroenterol. Hepatol. 2019, 8, 369. 31108231 10.1016/j.jcmgh.2019.05.003 PMC6718943

[26] Neunlist M, Toumi F, Oreschkova T, Denis M, Leborgne J, Laboisse CL, Galmiche J-P, Jarry A, Am. J. Physiol. 2003, 285, G1028. 10.1152/ajpgi.00066.2003 12881224

[27] Farack UM, Reiter J, Gross M, Moroder L, Wunsch E, Loeschke K, Scand. J. Gastroenterol. Suppl. 1987, 139, 32. 3481117 10.3109/00365528709089772

[28] Keita AV, Soderholm JD, Ericson AC, Neurogastroenterol. Motil 2010, 22, 770. 20149111 10.1111/j.1365-2982.2010.01471.x

[29] Middelhoff M, Nienhüser H, Valenti G, Maurer HC, Hayakawa Y, Takahashi R, Kim W, Jiang Z, Malagola E, Cuti K, Tailor Y, Zamechek LB, Renz BW, Quante M, Yan KS, Wang TC, Nat. Commun. 2020, 11, 111. 31913277 10.1038/s41467-019-13850-7 PMC6949263

[30] Huh D, Kim HJ, Fraser JP, Shea DE, Khan M, Bahinski A, Hamilton GA, Ingber DE, Nat. Protoc. 2013, 8, 2135. 24113786 10.1038/nprot.2013.137

[31] Ma C, Peng Y, Li H, Chen W, Trends Pharmacol. Sci. 2021, 42, 119. 33341248 10.1016/j.tips.2020.11.009 PMC7990030

[32] Soucy JR, Bindas AJ, Brady R, Torregrosa T, Denoncourt CM, Hosic S, Dai G, Koppes AN, Koppes RA, Adv. Biosyst. 2020, 4, 2000133. 10.1002/adbi.202000133 PMC8136149 32755004

[33] Hayes JA, Lunger AW, Sharma AS, Fernez MT, Carrier RL, Koppes AN, Koppes R, Woolston BM, Cell Rep. 2023, 42, 113481. 37980564 10.1016/j.celrep.2023.113481 PMC10791167

[34] Soucy JR, Burchett G, Brady R, Brady R, Nichols K, Breault DT, Koppes AN, Koppes RA, Organs Chip 2021, 3, 100009. 38650595 10.1016/j.ooc.2021.100009 PMC11034938

[35] Barker N, Nat. Rev. Mol. Cell Biol. 2014, 15, 19. 24326621 10.1038/nrm3721

[36] Darwich AS, Aslam U, Ashcroft DM, Drug Metab. Dispos. 2014, 42, 2016. 25233858 10.1124/dmd.114.058404

[37] Williams JM, Duckworth CA, Burkitt MD, Watson AJ, Campbell BJ, Pritchard DM, Vet. Pathol. 2015, 52, 445. 25428410 10.1177/0300985814559404 PMC4441880

[38] Lentle RG, Janssen PW, J. Comp. Physiol. B 2008, 178, 673. 18401586 10.1007/s00360-008-0264-x

[39] Ishikawa T, Sato T, Mohit G, Imai Y, Yamaguchi T, J. Theor. Biol. 2011, 279, 63. 21440560 10.1016/j.jtbi.2011.03.026

[40] Poon C, J. Mech. Behav. Biomed. Mater. 2022, 126, 105024. 34911025 10.1016/j.jmbbm.2021.105024

[41] Zhou X, Liu D, You L, Wang L, Biomech J. 2010, 43, 1598. 10.1016/j.jbiomech.2009.12.028 PMC2866761 20185133

[42] Venkatraman A, Yu W, Nitkin C, Sampath V, Cells. 2021, 10, 312. 33546361 10.3390/cells10020312 PMC7913590

[43] Noorian AR, Taylor GM, Annerino DM, Greene JG, J. Comp. Neurol. 2011, 519, 3387. 21618236 10.1002/cne.22679 PMC4033411

[44] Brehmer A, Histochem. Cell Biol. 2021, 156, 95. 34170401 10.1007/s00418-021-02002-y PMC8397665

[45] Parathan P, Wang Y, Leembruggen AJ, Bornstein JC, Foong JP, Dev. Biol. 2020, 458, 75. 31629713 10.1016/j.ydbio.2019.10.011

[46] Chevalier NR, Gazquez E, Bidault L, Guilbert T, Vias C, Vian E, Watanabe Y, Muller L, Germain S, Bondurand N, Dufour S, Fleury V, Sci. Rep. 2016, 6, 20927. 26887292 10.1038/srep20927 PMC4757826

[47] Allen M, Ghosh S, Ahern GP, Villapol S, Maguire-Zeiss KA, Conant K, Sci. Rep. 2016, 6, 35497. 27762280 10.1038/srep35497 PMC5071868

[48] Puzan M, Hosic S, Ghio C, Koppes A, Sci. Rep. 2018, 8, 1. 29679034 10.1038/s41598-018-24768-3 PMC5910425

[49] Yang D, Jacobson A, Meerschaert KA, Sifakis JJ, Wu M, Chen X, Yang T, Zhou Y, Anekal PV, Rucker RA, Sharma D, Sontheimer-Phelps A, Wu GS, Deng L, Anderson MD, Choi S, Neel D, Lee N, Kasper DL, Jabri B, Huh JR, Johansson M, Thiagarajah JR, Riesenfeld SJ, Chiu IM, Cell 2022, 185, 4190. 36243004 10.1016/j.cell.2022.09.024 PMC9617795

[50] Van Landeghem L, Chevalier J, Mahé MM, Wedel T, Urvil P, Derkinderen P, Savidge T, Neunlist M, Am. J. Physiol. 2011, 300, G976. 10.1152/ajpgi.00427.2010 PMC3119120 21350188

[51] Yu YB, Li YQ, World J Gastroenterol. 2014, 20, 11273. 10.3748/wjg.v20.i32.11273 PMC4145765 25170211

[52] Le Gall SM, Meneton P, Mauduit P, Dreux C, Regul. Pept. 2004, 122, 119. 15380929 10.1016/j.regpep.2004.06.008

[53] Holloway EM, Czerwinski M, Tsai Y-H, Wu JH, Wu A, Childs CJ, Walton KD, Sweet CW, Yu Q, Glass I, Treutlein B, Camp JG, Spence JR, Cell Stem Cell 2021, 28, 568. 33278341 10.1016/j.stem.2020.11.008 PMC7935765

[54] Abud HE, Chan WH, Jarde T, Front Cell Dev. Biol. 2021, 9, 685665. 34350179 10.3389/fcell.2021.685665 PMC8327171

[55] Zou WY, Blutt SE, Zeng X-L, Chen M-S, Lo Y-H, Castillo-Azofeifa D, Klein OD, Shroyer NF, Donowitz M, Estes MK, Cell Rep. 2018, 22, 1003. 29386123 10.1016/j.celrep.2017.12.093 PMC5798462

[56] Liu X, Lu R, Wu S, Zhang Y-G, Xia Y, Sartor BR, Sun J, Inflamm. Bowel Dis. 2012, 18, 418. 21674728 10.1002/ibd.21788 PMC3294455

[57] Jung YS, Jun S, Lee SH, Sharma A, Park JI, OncoTargets Ther. 2015, 6, 37257. 10.18632/oncotarget.6133 PMC4741928 26484565

[58] Pearce SC, Al-Jawadi A, Kishida K, Yu S, Hu M, Fritzky LF, Edelblum KL, Gao N, Ferraris RP, BMC Biol. 2018, 16, 19. 29391007 10.1186/s12915-018-0481-z PMC5793346

[59] Pope JL, Bhat AA, Sharma A, Ahmad R, Krishnan M, Washington MK, Beauchamp RD, Singh AB, Dhawan P, Gut 2014, 63, 622. 23766441 10.1136/gutjnl-2012-304241 PMC4083824

[60] Sato T, Clevers H, Science 2013, 340, 1190. 23744940 10.1126/science.1234852

[61] Kwon O, Yu WD, Son YS, Jung KB, Lee H, Son MY, Int. J. Stem Cells 2022, 15, 104. 35220296 10.15283/ijsc21209 PMC8889332

[62] Nguyen HD, Aljamaei HM, Stadnyk AW, Cell Mol. Gastroenterol. Hepatol. 2021, 12, 1343. 34271223 10.1016/j.jcmgh.2021.07.005 PMC8463866

[63] Puzan M, Hosic S, Ghio C, Koppes A, Sci. Rep. 2018, 8, 6313. 29679034 10.1038/s41598-018-24768-3 PMC5910425

[64] Bornstein JC, Foong JP, in Physiology of the Gastrointestinal Tract, Elsevier, Amsterdam 2018, p. 429.

[65] Poddar D, Kaur R, Baldwin WM 3rd, Mazumder B, Cell Mol. Immunol. 2016, 13, 816. 26166763 10.1038/cmi.2015.53 PMC5101439

[66] Ruder B, Atreya R, Becker C, Int. J. Mol. Sci. 2019, 20, 1887. 30995806 10.3390/ijms20081887 PMC6515381

[67] Dheer R, Santaolalla R, Davies JM, Lang JK, Phillips MC, Pastorini C, Vazquez-Pertejo MT, Abreu MT, Infect. Immun. 2016, 84, 798. 26755160 10.1128/IAI.01374-15 PMC4771346

[68] Hayden MS, Ghosh S, in Regulation of NF-κB by TNF Family Cytokines, Elsevier, Amsterdam 2014, pp. 253–266. 10.1016/j.smim.2014.05.004 PMC4156877 24958609

[69] Lamouille S, Xu J, Derynck R, Nat. Rev. Mol. Cell Biol. 2014, 15, 178. 24556840 10.1038/nrm3758 PMC4240281

[70] Leggett SE, Hruska AM, Guo M, Wong IY, Cell Commun. Signal. 2021, 19, 32. 33691719 10.1186/s12964-021-00713-2 PMC7945251

[71] Liu X, Wang X, Zhang L, Sun L, Wang H, Zhao H, Zhang Z, Liu W, Huang Y, Ji S, Zhang J, Li K, Song B, Li C, Zhang H, Li S, Wang S, Zheng X, Gu Q, Adv. Healthc. Mater. 2021, 10, 2101405. 10.1002/adhm.202101405 34634194

[72] Azman M, Sabri AH, Anjani QK, Mustaffa MF, Hamid KA, Pharmaceuticals 2022, 15. 10.3390/ph15080975 PMC9412385 36015123

[73] Zhang J, Huang Y-J, Yoon JY, Kemmitt J, Wright C, Schneider K, Sphabmixay P, Hernandez-Gordillo V, Holcomb SJ, Bhushan B, Rohatgi G, Benton K, Carpenter D, Kester JC, Eng G, Breault DT, Yilmaz O, Taketani M, Voigt CA, Carrier RL, Trumper DL, Griffith LG, Med 2021, 2, 74. 33511375 10.1016/j.medj.2020.07.001 PMC7839961

[74] Sontheimer-Phelps A, Chou DB, Tovaglieri A, Ferrante TC, Duckworth T, Fadel C, Frismantas V, Sutherland AD, Jalili-Firoozinezhad S, Kasendra M, Stas E, Weaver JC, Richmond CA, Levy O, Prantil-Baun R, Breault DT, Ingber DE, Cell Mol. Gastroenterol. Hepatol. 2020, 9, 507. 31778828 10.1016/j.jcmgh.2019.11.008 PMC7036549

[75] Wang Y, Sims CE, Allbritton NL, Anal. Chem. 2022, 94, 9345. 35736812 10.1021/acs.analchem.2c00905 PMC9337237

[76] Schindelin J, Arganda-Carreras I, Frise E, Kaynig V, Longair M, Pietzsch T, Preibisch S, Rueden C, Saalfeld S, Schmid B, Tinevez J-Y, White DJ, Hartenstein V, Eliceiri K, Tomancak P, Cardona A, Nat. Methods 2012, 9, 676. 22743772 10.1038/nmeth.2019 PMC3855844

[77] Legland D, Arganda-Carreras I, Andrey P, Bioinformatics 2016, 32, 3532. 27412086 10.1093/bioinformatics/btw413

[78] Stirling DR, Swain-Bowden MJ, Lucas AM, Carpenter AE, Cimini BA, Goodman A, BMC Bioinform. 2021, 22, 1. 10.1186/s12859-021-04344-9 PMC8431850 34507520

[79] Ge SX, Son EW, Yao R, BMC Bioinform. 2018, 19, 1. 10.1186/s12859-018-2486-6 PMC6299935 30567491

[80] Jassal B, Matthews L, Viteri G, Gong C, Lorente P, Fabregat A, Sidiropoulos K, Cook J, Gillespie M, Haw R, Loney F, May B, Milacic M, Rothfels K, Sevilla C, Shamovsky V, Shorser S, Varusai T, Weiser J, Wu G, Stein L, Hermjakob H, D’ Eustachio P, Nucleic Acids Res. 2020, 48, D498. 31691815 10.1093/nar/gkz1031 PMC7145712

