# Supplementary material for: A Pumpless, High-Throughput Microphysiological System to Mimic Enteric Innervation of Duodenal Epithelium and the Impact on Barrier Function
Source: Adv Funct Mater. Author manuscript; Available in PMC 2025 Aug 27. (PMC12380100; doi:10.1002/adfm.202409718)
Supplement: Supp Material [file NIHMS2086014-supplement-Supp_Material.pdf]

ADVANCED FUNCTIONAL MATERIALS

Supporting Information

for *Adv. Funct. Mater.*, DOI 10.1002/adfm.202409718

A Pumpless, High-Throughput Microphysiological System to Mimic Enteric Innervation of Duodenal Epithelium and the Impact on Barrier Function

Kyla N. Kaiser, Jessica R. Snyder, Ryan A. Koppes and Abigail N. Koppes**

A pumpless, high-throughput microphysiological system confirms enteric innervation of duodenal epithelium strengthens barrier function

Kyla N. Kaiser[1,#], Jessica R. Snyder[2,#], Ryan A. Koppes[1,2*], Abigail N. Koppes[1,2,3*]

1. Department of Chemical Engineering, Northeastern University, Boston, MA
2. Department of Bioengineering, Northeastern University, Boston, MA
3. Department of Biology, Northeastern University, Boston, MA

*Corresponding Authors: Ryan Koppes r.koppes@northeastern.edu; Abigail Koppes a.koppes@northeastern.edu

# authors contributed equally to this work

Supplemental Materials

Table S1: Enteric neuron medium composition

Medium Reagent	Supplier/Catalog #	Final Concentration
Neurobasal Medium	Gibco, 10888022	97%
Fetal Bovine Serum	Corning, 35015CV	1%
Antibiotic/Antimycotic	Gibco, 15240-062	1%
Glutamax	Gibco, 35050061	1X
B27 Supplement	Gibco, 17504044	1X
GDNF	Gibco, PHC7045	10ng/mL
NGF	R&D Systems, 256-GF/CF	25ng/mL

Table S2: Expansion medium composition

Medium Reagent	Supplier & Catalog #	Working Concentration	Final Concentration	Volume for 50 mL
WRN Media	HDDC Organoid Core/Breault Lab	N/A	50%	25 mL
Adv DMEM/F12	Gibco, 12634028	N/A	45%	22.5 mL
Glutamax	Gibco, 35050061	100X	1X	500 µL
HEPES	Fisher, AAJ16924AE	1 M	10 mM	500 µL
Primocin	Invivogen, NC9141851	50 mg/mL	100 µg/mL	100 µL
Normocin	Invivogen, NC9273499	50 mg/mL	100 µg/mL	100 µL
B27	Gibco, 17504-044	50X	0.5X	500 µL
N2	Gibco, A13707-01	100X	0.5X	250 µL
N-Acetyl-Cysteine	Sigma, A7250	500 mM	500 µM	50 µL
A-83-01	Sigma, SML0788	500 µM	500 nM	50 µL
SB202190	Sigma, S7067	30 mM	10 µM	16.6 µL
EGF	Peprotech, 315-09	50 µg/mL	5 ng/mL	5 µL
Gastrin	Sigma, G9145	100 µM	10 nM	5 µL
Y-27632 (only added after passaging)	Tocris Bioscience, 12-541-0	10 mM	10 µM	50 µL

Table S3: Epithelial differentiation medium composition

Medium Reagent	Supplier & Catalog #	Working Concentration	Final Concentration	Volume for 10 mL
Advanced DMEM/F12	Gibco, 12634028	N/A	85%	8.5 mL
R-Spondin	HDDC Organoid Core/Breault Lab	N/A	10%	1 mL
Glutamax	Gibco, 35050061	100X	1X	100 µL
HEPES	Fisher, AAJ16924AE	1M	10 mM	100 µL
Primocin	Invivogen, NC9141851	50 mg/mL	100 µg/mL	20 µL
Normocin	Invivogen, NC9273499	50 mg/mL	100 µg/mL	20 µL
B27 Supplement	Gibco, 17504-044	50X	0.5X	100 µL
N2 Supplement	Gibco, A13707-01	100X	0.5X	50 µL
N-Acetyl-Cysteine	Sigma, A7250	500 mM	1.25 mM	10 µL
EGF	Peprotech, 315-09	50 µg/mL	5 ng/mL	1 µL
Noggin	Peprotech, 250-38	100 µg/mL	100 ng/mL	10 µL
Y-27632 (Rock Inhibitor)	Sigma, Y0503	10 mM	10 uM	10 µL
A-83-01	Sigma, SML0788	500 µM	500 nM	10 µL
SB202190	Sigma, S7067	30 mM	10 µM	3.3 µL
Gastrin	Sigma, G9145	100 µM	10 nM	1 µL
Valproic Acid	Sigma, P4543	1 M	1 mM	10 µL
CHIR99021	Sigma, SML1046	10 mM	3 µM	3 µL

Table S4: Antibodies and dilutions used for immunocytochemistry

Antibody	Catalog ID/Supplier	Host	ICC Dilution	Notes
Beta III Tubulin	Sigma, T8660	Mouse	1:1000	Structural, mature neurons<br>Lot #s: 0000139791, 0000116046
Zonula Occludens-1 (ZO-1)	Invitrogen, 40-2200	Rabbit	1:100-1:200	Tight junctions, mature epithelium<br>Lot #: UE283841
Vasoactive Intestinal Peptide (VIP)	Abcam, AB8556	Rabbit	1:200	VIP producing neurons<br>Lot #: GR236115-1
Choline Acetyltransferase (ChAT)	Millipore, AB15468	Chicken	1:1000	Acetylcholine producing neurons<br>Lot #: 3156103
Phalloidin Alexa Fluor 647	Invitrogen, A22287	NA	1:1000	F-actin
Anti-Chicken, Alexa Fluor 488	Invitrogen, A11039	Goat	1:1000	Secondary
Anti-Rabbit, Alexa Fluor 546	Invitrogen, A11035	Goat	1:1000	Secondary
Anti-Mouse, Alexa Fluor 647	Invitrogen, A32728	Goat	1:1000	Secondary

Table S5: Relative expression (Log_2(CPM+c)) from RNA sequencing for genes of interest in freshly isolated duodenal tissue from neonatal rats, primary epithelial cells, and primary epithelial cells co-cultured with neurons, separated by a membrane.

Gene ID	Duodenum Tissue		Monocultured Epithelium		Co-cultured Epithelium	
**WNT Genes of Interest**						
wnt2	5.47	4.68	2.00	2.00	2.58	2.63
wnt6	2.49	2.36	2.00	5.16	2.00	4.79
wnt3	4.97	7.13	5.25	5.22	3.98	5.20
wnt9b	9.78	9.27	4.03	4.59	2.98	3.77
**Proliferation and Tight Junction Formation Genes Of Interest**						
lgr5	6.58	10.86	6.93	6.78	6.67	6.07
mki67	12.35	13.59	10.53	11.57	10.12	10.86
vil1	15.07	14.78	13.26	13.10	12.99	13.44
tjp1	12.61	12.42	13.47	14.22	13.95	14.66
ocln	11.58	12.06	11.91	11.94	12.05	12.65
cldn1	7.14	6.24	9.76	11.60	10.35	12.80

cldn3	7.32	9.19	4.46	11.20	4.43	11.36
**Mucin Genes of Interest**						
muc2	15.83	15.90	12.67	10.64	12.49	10.94
muc6	8.42	10.98	6.99	7.74	6.64	7.22
muc1	7.72	9.49	11.05	9.63	12.21	8.79
muc13	8.82	9.69	5.63	10.35	6.14	10.59
muc5b	11.86	13.09	11.78	4.44	11.85	5.36
muc15	3.38	2.88	4.69	3.89	3.98	3.51
**Inflammatory Genes of Interest**						
il18	10.60	10.28	4.11	6.98	4.78	7.88
il10	3.14	2.51	2.00	3.55	2.98	6.20
il1b	6.43	4.63	5.46	7.41	5.61	8.75
tnfa	3.38	2.35	7.31	7.78	7.40	6.66
tlr4	4.21	3.26	6.30	7.45	6.52	8.16
**Neuronal Genes of Interest**						
ncam2	4.81	4.38	2.00	2.00	2.00	4.83
st6galnac5	4.33	4.32	2.00	2.00	2.58	3.84
slc2a	7.55	6.70	2.00	2.00	2.00	2.94
grik2	2.00	2.64	2.00	2.47	2.00	4.29
tac1	5.18	6.01	3.65	6.19	5.29	9.23
**Acetylcholine and VIP Receptor Genes of Interest**						
chrm3	8.84	9.53	9.19	9.84	9.00	9.74
chrna10	2.49	4.78	2.00	3.64	2.00	2.25
vipr2	6.28	8.47	3.85	3.64	2.57	3.42

Table S6: Top 20 enriched Reactome pathways with the number of genes and normalized enrichment scores for co-cultured versus monocultured epithelial cells.

Pathway	# of Genes	Normalized Enrichment Score (NES)
L13a-mediated translational silencing of Ceruloplasmin expression	91	2.7521
GTP hydrolysis and joining of the 60S ribosomal subunit	92	2.7313
Eukaryotic Translation Initiation	98	2.7005
Cap-dependent Translation Initiation	98	2.7005
Formation of a pool of free 40S subunits	82	2.6973

SRP-dependent cotranslational protein targeting to membrane	76	2.6829
Nonsense Mediated Decay (NMD) independent of the Exon Junction Complex (EJC)	78	2.6539
Respiratory electron transport, ATP synthesis by chemiosmotic<br>coupling, and heat production by uncoupling proteins.	94	2.562
Nonsense-Mediated Decay (NMD)	93	2.5414

Table S7: Top 100 most differentially expressed genes between co-cultured epithelium with myenteric neurons and epithelium monocultures from RNA sequencing based on log2 fold change. Epithelium monocultures and co-cultures compared to freshly isolated duodenum epithelium were also included.

Ensembl ID	GENE ID	EPI+EN vs EPI (log2 fold change)	EPI vs Isolated Duodenum (log2 fold change)	EPI+EN vs Isolated Duodenum (log2 fold change)
ENSRNOG00000002126	Ncam2	6.28603	-7.00833	-0.7223
ENSRNOG00000051537	AABR07006673.1	5.689542	-3.83826	1.851285
ENSRNOG00000058052	AABR07060915516.1	5.391814	-5.77947	-0.38765
ENSRNOG00000049676	St6galnac5	5.326859	-6.66811	-1.34125
ENSRNOG00000062950	U13	5.230234	-1.56835	3.661885
ENSRNOG00000057709	Snord8	4.847672	-4.56659	0.281085
ENSRNOG00000052487	Snord73	4.65328	-4.66943	-0.01615
ENSRNOG00000004302	Pah	4.607771	-6.92297	-2.31519
ENSRNOG00000029191	LOC685067	4.545121	-8.69259	-4.14747
ENSRNOG00000063461	U2	4.524145	-5.73161	-1.20747
ENSRNOG00000069609	NA	4.455111	-8.14492	-3.68981
ENSRNOG00000067735	NA	4.295336	-5.46307	-1.16773
ENSRNOG00000055009	U6	4.257838	-4.18448	0.073356
ENSRNOG00000006604	Thy1	4.15657	-6.32108	-2.16451
ENSRNOG00000049826	UST4r	4.126229	-9.07052	-4.94429
ENSRNOG00000066167	NA	4.097251	-6.15354	-2.05629

ENSRNOG00000019810	Des	3.979562	-7.85227	-3.8727
ENSRNOG00000069670	Ugt2b	3.840931	-5.85886	-2.01793
ENSRNOG00000008376	Slc2a3	3.821664	-9.80787	-5.98621
ENSRNOG00000068634	Csta	3.779134	0.903222	4.682356
ENSRNOG00000051905	Wnt2	3.736891	-7.60572	-3.86882
ENSRNOG00000065655	NA	3.731947	2.167579	5.899526
ENSRNOG00000037549	AABR07058124.2	3.730844	-5.89787	-2.16702
ENSRNOG00000068803	U4	3.724455	-2.67051	1.053943
ENSRNOG00000068992	NA	3.716936	-4.60516	-0.88822
ENSRNOG00000035617	Mir186	3.658353	-3.54045	0.1179
ENSRNOG00000053017	AC131411.2	3.634987	-6.15361	-2.51862
ENSRNOG00000004704	Dcstamp	3.513132	3.482746	6.995877
ENSRNOG00000006579	Reg3g	3.49339	3.984277	7.477668
ENSRNOG00000027380	Upk1b	3.475379	-3.85933	-0.38395
ENSRNOG00000004900	Crhr1	3.38904	-8.71469	-5.32565
ENSRNOG00000060046	AABR07029664.1	3.383462	-7.64743	-4.26396
ENSRNOG00000069254	Cd209al1	3.383331	-9.26983	-5.8865
ENSRNOG00000000368	Grik2	3.318578	-0.58012	2.738456
ENSRNOG00000059833	AABR07073270.1	3.306823	-5.85931	-2.55248
ENSRNOG00000002829	Ppbp	3.256119	1.482059	4.738178
ENSRNOG00000004647	Il10	3.239131	0.316201	3.555333
ENSRNOG00000057127	Zg16b	3.232758	-4.96221	-1.72945
ENSRNOG00000000053	Crp	3.223745	-5.86881	-2.64507
ENSRNOG00000020953	Ms4a7	3.212911	-1.26288	1.950036
ENSRNOG00000066140	LOC120095795	3.158616	-0.59835	2.560269
ENSRNOG00000052986	U6	3.144266	-4.3284	-1.18413
ENSRNOG00000007137	Ly6i	3.099948	0.516732	3.61668
ENSRNOG00000019159	Jakmip2	3.066593	-0.72469	2.3419
ENSRNOG00000021364	LOC691311	3.033307	-7.77248	-4.73917

ENSRNOG00000067502	Tac1	3.023527	-0.25245	2.771075
ENSRNOG00000060922	AC118127.1	3.019049	-2.96095	0.058095
ENSRNOG00000013954	Alpl	3.007982	-1.24523	1.762756
ENSRNOG00000006726	Zfp9	2.98044	-5.19878	-2.21834
ENSRNOG00000004679	Fign	2.97647	-7.07128	-4.09481
ENSRNOG00000070284	NA	-2.90871	1.580207	-1.3285
ENSRNOG00000071185	NA	-2.95488	-1.34425	-4.29914
ENSRNOG00000010047	Ddit4l2	-2.96854	1.026878	-1.94166
ENSRNOG00000070087	NA	-2.97473	-0.32485	-3.29959
ENSRNOG00000020293	Chrna10	-2.97802	-1.55411	-4.53213
ENSRNOG00000062856	NA	-2.98052	-7.94207	-10.9226
ENSRNOG00000066888	NA	-2.98194	5.089741	2.107799
ENSRNOG00000017206	Igfbp5	-2.99046	0.523919	-2.46654
ENSRNOG00000021048	AC095693.1	-3.011	2.258104	-0.75289
ENSRNOG00000002501	Ddx3y	-3.03803	-5.46497	-8.503
ENSRNOG00000033654	LOC501038	-3.07014	-5.6026	-8.67275
ENSRNOG00000051169	Clnk	-3.07234	-5.46025	-8.53258
ENSRNOG00000003305	Cxcr3	-3.07541	-5.21188	-8.2873
ENSRNOG00000068920	NA	-3.08279	2.981073	-0.10172
ENSRNOG00000002947	Dpt	-3.09116	-5.56348	-8.65464
ENSRNOG00000048771	RGD1559482	-3.2057	-3.82111	-7.02681
ENSRNOG00000029811	Kcne2	-3.21567	1.292005	-1.92367
ENSRNOG00000064997	NA	-3.28077	-3.05507	-6.33584
ENSRNOG00000011754	Myom2	-3.30485	3.992981	0.688135
ENSRNOG00000050922	Nupr1l1	-3.30587	3.148077	-0.15779
ENSRNOG00000010906	Ccl5	-3.44644	-5.42981	-8.87625
ENSRNOG00000053034	AABR07071198.1	-3.46559	-0.90075	-4.36635
ERCC-00017	NA	-3.50119	4.303664	0.802473
ENSRNOG00000062833	NA	-3.50242	-2.00071	-5.50313

ENSRNOG00000046959	Mnx1	-3.51529	-2.52339	-6.03868
ENSRNOG00000069077	NA	-3.54245	-5.67	-9.21245
ENSRNOG00000068399	Itgad	-3.55515	-3.32618	-6.88133
ENSRNOG00000062675	NA	-3.56321	2.69938	-0.86383
ENSRNOG00000019216	Il12rb1	-3.60598	-2.59732	-6.20331
ENSRNOG00000069352	Aqp10	-3.61344	-5.27251	-8.88595
ENSRNOG00000003669	Myocd	-3.68294	-4.31504	-7.99798
ENSRNOG00000055796	AC103574.1	-3.68456	-2.85835	-6.54291
ENSRNOG00000019194	Pitx3	-3.78662	5.333423	1.5468
ENSRNOG00000069226	NA	-3.78912	5.762244	1.973122
ENSRNOG00000019265	Pcdh12	-3.93002	-3.28648	-7.21651
ENSRNOG00000011946	Ptn	-3.95726	-6.02338	-9.98064
ENSRNOG00000059326	Abca9	-3.96892	1.28982	-2.6791
ENSRNOG00000007310	Klrb1b	-3.97963	-5.78911	-9.76874
ENSRNOG00000009227	Aplnr	-4.02781	-5.33459	-9.3624
ENSRNOG00000023285	Akr1b1-ps2	-4.10603	4.475498	0.369467
ENSRNOG00000053624	AABR07058017.2	-4.11829	-2.76894	-6.88723
ENSRNOG00000068307	Rn18s	-4.14182	0.206428	-3.9354
ENSRNOG00000052173	AABR07008439.1	-4.33103	4.344374	0.013339
ENSRNOG00000065941	NA	-4.33935	5.973977	1.63463
ERCC-00098	NA	-4.40124	-2.21141	-6.61265
ENSRNOG00000049289	Robo4	-4.53504	-3.80765	-8.34269
ENSRNOG00000068222	LOC100910657	-4.7402	5.709368	0.969169
ENSRNOG00000042455	Tlr12	-4.93478	-3.21836	-8.15314
ENSRNOG00000031486	AABR07059215.1	-5.71052	6.060307	0
ENSRNOG00000064666	NA	-6.12789	2.615695	-3.51219

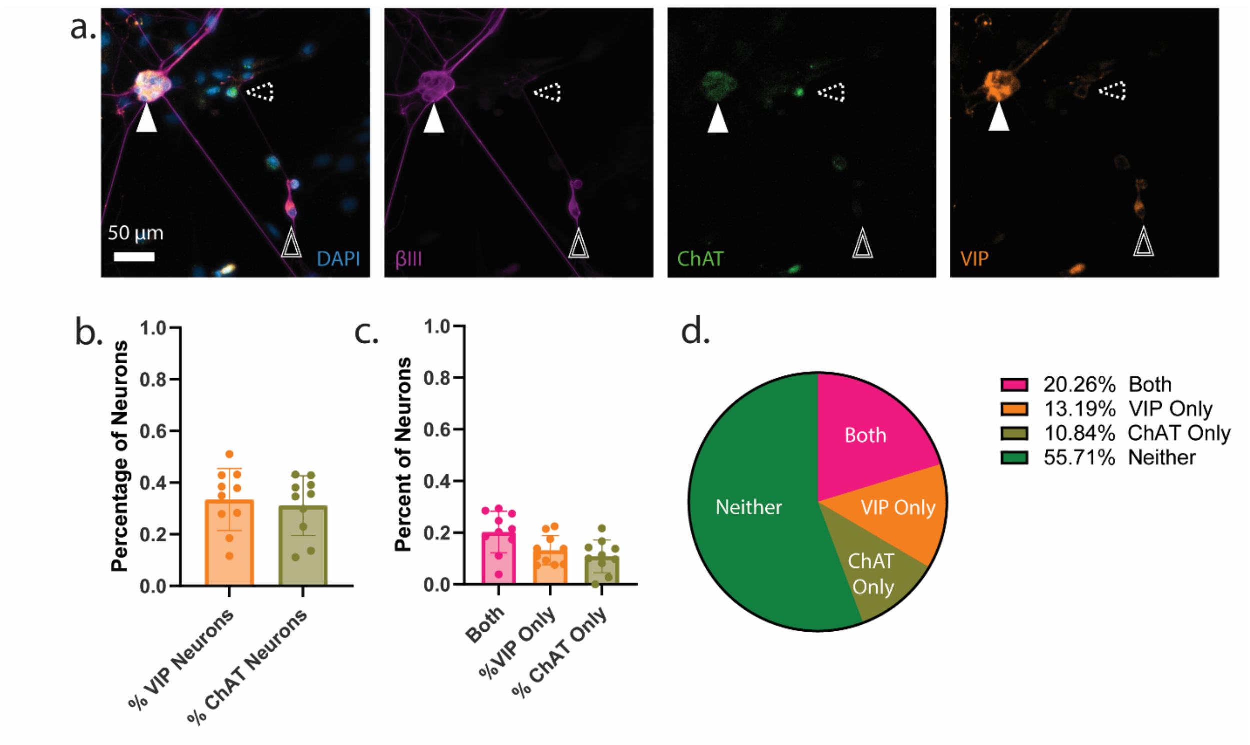

Figure S1: (a) Immunocytochemistry images of rat neonatal enteric neurons after 7 days under standard culture conditions. Cultures contained a heterogenous population of VIPergic (VIP, double outlined arrow), cholinergic (ChAT, dashed arrow), and co-expressing neurons (solid white arrow). (b) About 30% of all the neurons imaged expressed VIP and ChAT. (c, d) Many neurons expressed both VIP and ChAT in the cultures. Technical Replicates=3 (pooled tissue samples between 10 animals, mixed sex), wells per replicate=3-4, error bars = SD.

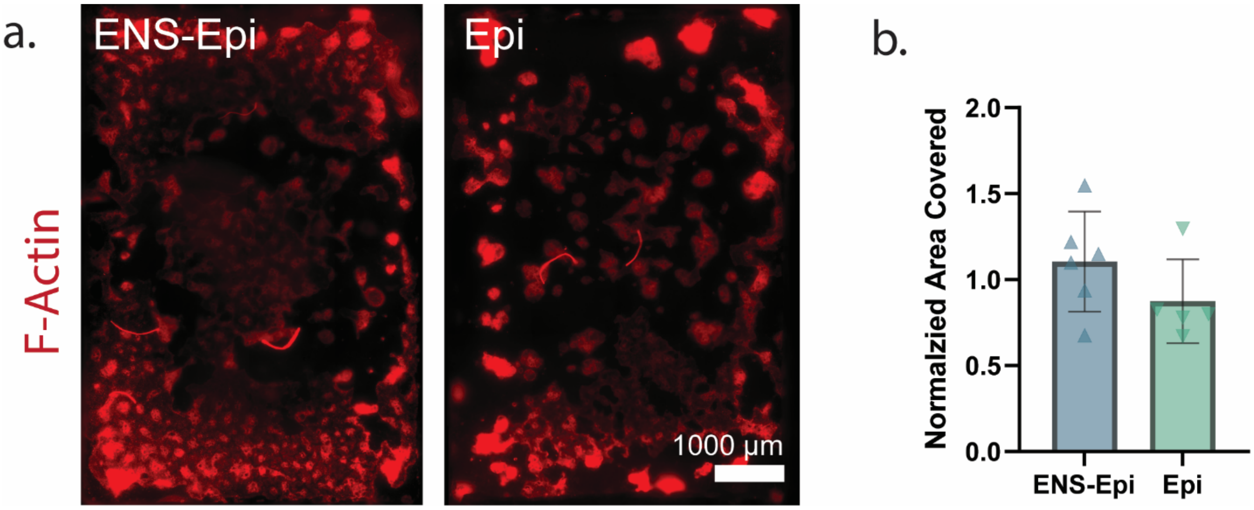

Figure S2: a. F-actin stained top view images of MPS epithelium. B. The area covered normalized to an average of all samples in a replicate n. Co-culture condition had a trending higher normalized coverage within the culture area than the epithelium alone. (n=2, m=2-3, normalized to an average of all samples in a replicate n).

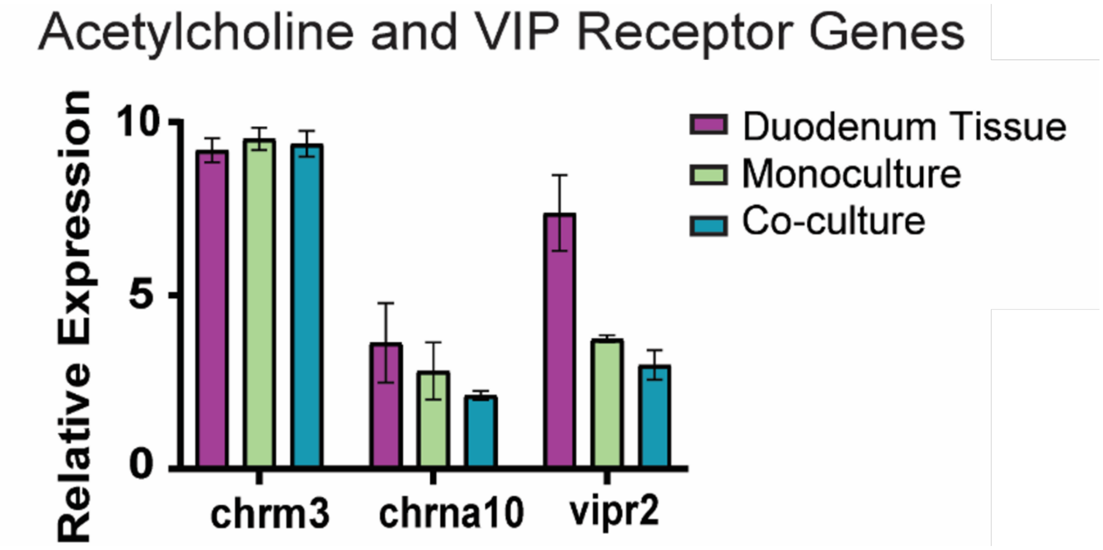

Figure S3: Relative gene expression of acetylcholine and VIP receptor genes from freshly isolated crypt tissue, epithelial monoculture, and epithelium from co-culture with enteric neurons. Analysis of n=2 of a pooled 3 samples, error bars = SEM.
